# An Integrated Study of Ramie (*Boehmeria nivea*), and Its Wild, Cultivated, and Feral Forms

**DOI:** 10.1002/ece3.71126

**Published:** 2025-03-20

**Authors:** Ying Zhao, Richard I. Milne, Jie Liu, Zhi‐Peng Li, Xiao‐Gang Fu, Ke Li, Amos Kipkoech, Zeng‐Yuan Wu

**Affiliations:** ^1^ Germplasm Bank of Wild Species, Kunming Institute of Botany Chinese Academy of Sciences Kunming Yunnan China; ^2^ State Key Laboratory for Conservation and Utilization of Bio‐Resources, Research Center of Perennial Rice Engineering and Technology, School of Agriculture Yunnan University Kunming Yunnan China; ^3^ Institute of Molecular Plant Sciences, School of Biological Sciences University of Edinburgh Edinburgh UK; ^4^ CAS Key Laboratory for Plant Diversity and Biogeography of East Asia, Kunming Institute of Botany Chinese Academy of Sciences Kunming Yunnan China; ^5^ University of Chinese Academy of Sciences Beijing China; ^6^ School of Ecology and Environmental Sciences Yunnan University Kunming Yunnan China

**Keywords:** *Boehmeria nivea*, ecology, feralization, morphology, phylogeography, taxonomy

## Abstract

Feralization, the re‐establishment of wild populations from domesticated ancestors, can involve multiple parallel character reversions and potentially also rampant gene flow with cultivated and/or naturally wild material. It hence poses great challenges for infraspecific classification, which may impede crop development, but studies on these issues are rare. Ramie ( *Boehmeria nivea* ; Urticaceae) is an important fiber crop worldwide. It has been traditionally divided into 2–4 varieties, but these are controversial. Here, 78 wild and feral individuals were sampled from 12 Chinese provinces, plus 11 cultivated individuals from farmland. We employed an integrative taxonomy approach combining multiple lines of evidence from morphology, phylogenomics, and ecology to investigate the intraspecific subdivision of *B. nivea* . A chi‐square test of qualitative morphological characters significantly distinguished three varieties within *B. nivea* : var. *nivea*, var. *tenacissima*, and the recently described var. *strigosa*, comprising mainly cultivated, mainly feralized, and only naturally wild material, respectively. The PCoA and random forest analyses indicated differences between var. *strigosa* and the other two varieties. However, quantitative characters could not distinguish the three varieties. None of the three varieties was monophyletic based on the phylogeny of plastome data, whereas var. *strigosa* was weakly supported as monophyletic based on nuclear ribosomal DNA (18S‐ITS1‐5.8S‐ITS2‐26S). Ecological niche modeling showed overlap between the potential distribution areas of var. *nivea* and var. *tenacissima*, but neither overlapped with var. *strigosa*. These analyses collectively demonstrate the distinctiveness of var. *strigosa*, but mostly did not fully separate var. *nivea* from var. *tenacissima*. Hence, var. *strigosa* is a biologically meaningful variety, but var. *tenacissima* should be synonymized within var. *nivea*. These results should aid the breeding and improvement of new varieties of ramie and highlight the value of integrative taxonomic methods in examining infraspecific subdivisions within species that include cultivated and feralized material.

## Graphical abstract

This study employed an integrative taxonomy approach to investigate the infraspecific classification of *Boehmeria nivea* (Ramie), a key fiber crop, analyzing 78 wild and feral individuals from 12 Chinese provinces and 11 cultivated ones. Morphological, phylogenomic, and ecological analyses revealed three varieties: var. *nivea*, var. *tenacissima* (mostly cultivated and feralized), and the distinct var. *strigosa* (exclusively wild), with the latter being ecologically unique and suggested to be a valid separate variety. The results recommend synonymizing var. *tenacissima* within var. *nivea* due to overlapping characteristics, aiding in the crop's breeding and highlighting the value of integrated taxonomic methods for plants with cultivated and feral origins.

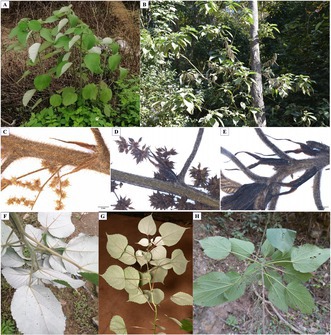

## 1 Introduction

Species subdivision (infraspecific diversity) has important implications for biological research and species conservation (De Queiroz 2007). However, infraspecific taxa are almost by definition partly interfertile, and hence hybridization and introgression between them can make their delimitation more difficult (Abbott et al. 2013; Harrison and Larson 2014). Infraspecific classification is further complicated by domestication, which often creates new anthropogenic varieties or subspecies, which may be defined by relatively few characters that are under strong selection and whose true relationship to wild forms remains uncertain (Purugganan and Fuller 2009; Meyer and Purugganan 2013).

Feralization is the naturalization or establishment of wild colonies or populations derived from domesticated material (Koch et al. 2024), and this may further complicate the taxonomic situation (Stitzer and Ross‐Ibarra 2018). Feral individuals may retain some characters acquired through domestication while other characters are altered by selective sweeps as they return to a wild environment (Pisias et al. 2022). Reliance solely on morphological character analysis or phylogenetic studies is inadequate to fully uncover the intricate relationships within feralized populations. Here we integrate information from morphological characters, multiple genomic datasets, and ecology to define the infraspecific classification of *B. nivea* .

*Boehmeria nivea* (L.) Gaudich. (Urticaceae), commonly known as ramie, is a perennial subshrub or shrub first described in 1830 (Gaudichaud‐Beaupré 1830) and consistently placed within *Boehmeria* in Clade I of Urticaceae (Wu et al. 2013; Wu et al. 2018). Ramie is historically significant as a fiber crop in Southeast and Eastern Asia, particularly in Japan, where archaeological evidence suggests it was introduced several thousand years ago (Liu et al. 2023), and it is mainly distributed in the montane regions of Southeast Asia, China, Korea, and Japan (GBIF 2024). Bast fiber extracted from the stem is strong and suitable for weaving fine textiles. Today, it is predominantly cultivated in China, especially in provinces across the south of the Yangtze River Basin (Chen et al. 2003) (Table S1; Figure S1), with well‐documented economic importance (Wang and Chen 1995; Chen et al. 2003; Hitchcock 2007; Roy and Lutfar 2012; Wang et al. 2019; Chew et al. 2022). Despite China's leading role in ramie production (Wang and Chen 1995; Chen et al. 2003; Banerjee et al. 2015), the planting area has seen a dramatic decrease of at least 50% over the past 50 years (Satya et al. 2019), leading to numerous abandoned fields and opportunities for feralization.

**FIGURE 1:**
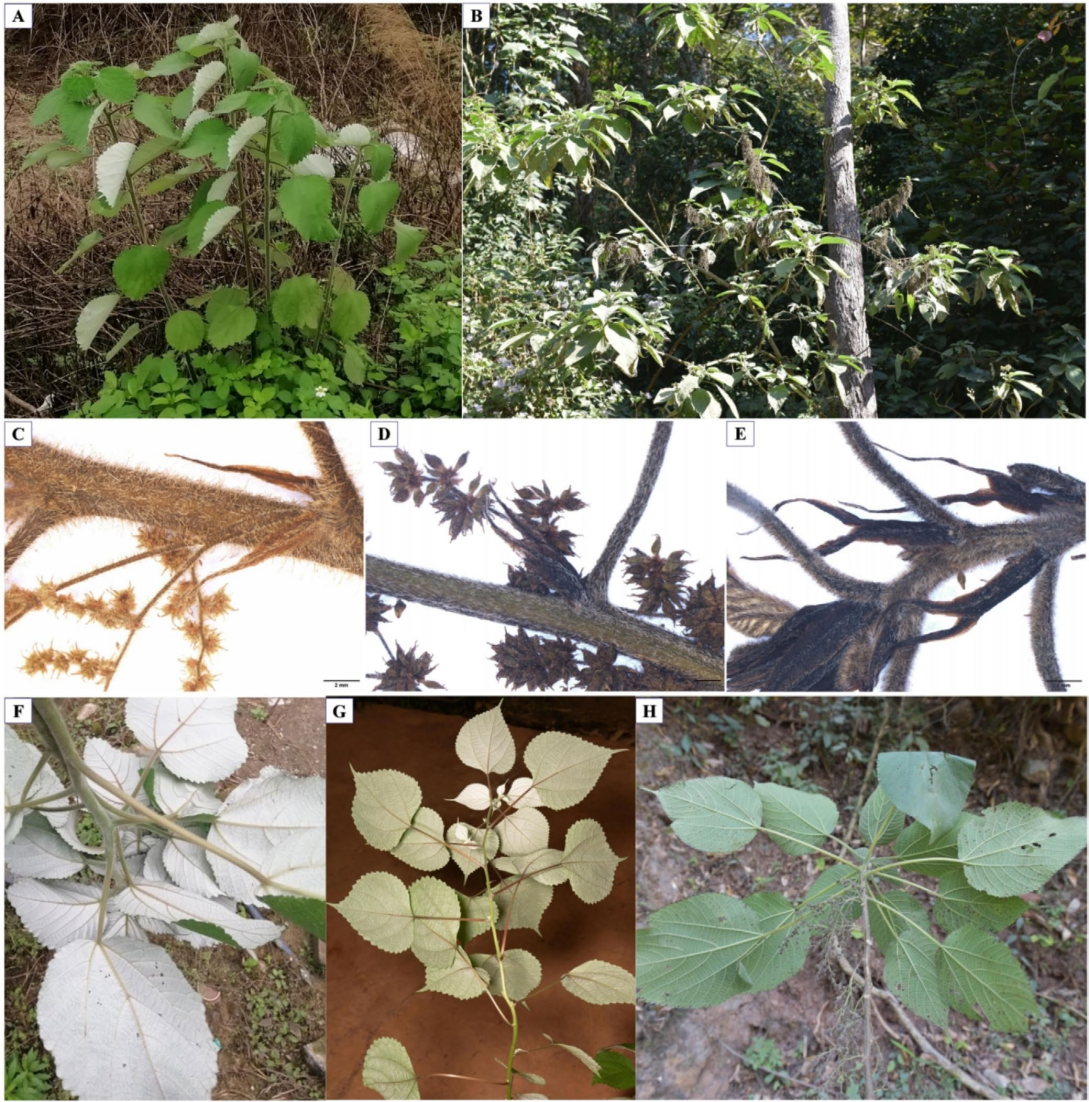
Morphological diversity of *Boehmeria nivea* . (A, B) habit: (A) *B. nivea* var. *nivea*, unbranched (collection number: Liuj2021225); (B) *B. nivea* var. *strigosa*, branched (collection number: WuZY2021573). (C–E) upper stems, petioles, and stipules, plus fruiting shoots in (C and D): (C) *B. nivea* var. *nivea*, densely patent hirsute with free stipules (collection number: Liuj14094); (D) *B. nivea* var. *tenacissima*, appressed strigose with partly connate stipules (collection number: WuZY2020714); (E) *B. nivea* var. *strigosa*, densely but shortly patently hirsute, with connate stipules (collection number: Wuzy20201080). (F–H) color of abaxial leaf blade: (F) *B. nivea* var. *nivea*, white (collection number: Liuj2021197); (G) *B. nivea* var. *tenacissima*, mixed (gray and white) (collection number: WuZY2020736); (H) *B. nivea* var. *strigosa*, green (collection number: WuZY2021571).

Key morphological characters useful in differentiating varieties in *B. nivea* include stems, petiole hair, leaf shape, stipule connateness, and the color of the abaxial leaf blade (Figure 1). Based on these characters, Wang and Chen (1995) recognized four varieties: var. *nivea*, var. *nipononivea* (Koidz.) W.T.Wang, var. *viridula* Yamam., and var. *tenacissima* (Roxb.) Miq. Subsequently, Chen et al. (2003) regarded var. *nipononivea* and var. *viridula* as synonyms of var. *tenacissima*, hence recognizing two varieties: var. *nivea* (stems and petioles densely patent hirsute, and stipules free; Figure 1C) and var. *tenacissima* (stems and petioles sparsely appressed strigose, with hairs occasionally dense and assurgent, stipules connate at the base, rarely to the middle; Figure 1D). In addition, our extensive fieldwork has revealed notable variations among populations in characters such as the leaf margin and leaf tip. The characteristics that supposedly distinguish var. *nivea* from var. *tenacissima*, according to Chen et al. (2003) were not always consistent in the field. This suggests the current criteria used for defining varieties may require reassessment (Table S2). Nevertheless, we found that *B. nivea* can be classified more consistently with the key of Chen et al. (2003) than with the key of Wang and Chen (1995).

Some individuals did not fit either var. *nivea* or var. *tenacissima*, exhibiting densely patent hirsute hairs on stems and petioles, partially connate stipules (Figure 1E), and a green abaxial leaf blade (Figure 1H). These individuals were consistent with the morphological characteristics of the recently described variety *B. nivea* var. *strigosa* Z. Y. Wu & Y. Zhao (Zhao et al. 2024). Recent studies on the domestication and feralization of ramie have also revealed the complexity of taxonomic relationships within ramie species (Wu et al. 2024). In the absence of genetic and other evidence, the distinctions between var. *nivea*, var. *tenacissima*, and var. *strigosa* remain unclear, impeding the understanding, conservation, and utilization of this plant.

To investigate the infraspecific classification of *B. nivea* , we conducted extensive field surveys, sampling, and genetic analysis to study the morphology, geographic distribution, habitat and ecological parameters, and phylogeography of this species and its varieties.

## 2 Materials and Methods

### 2.1 Taxon Sampling

**FIGURE 2:**
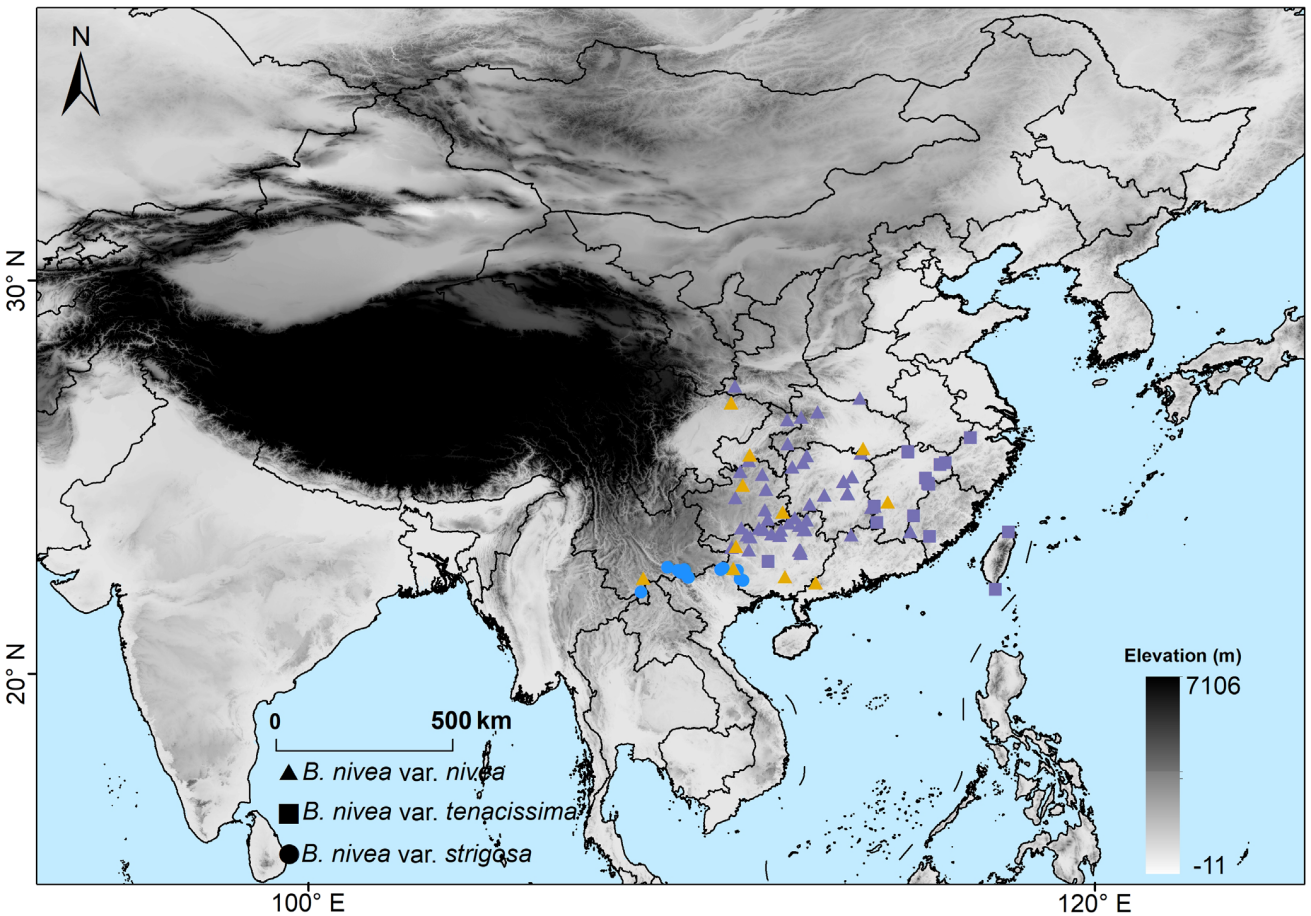
Sample distribution for *Boehmeria nivea* varieties (see key in figure): Blue, purple and yellow fill colors represent wild, feral, and domesticated individuals, respectively.

We sampled a total of 89 individuals of *B. nivea* during its flowering and fruiting seasons from August to December 2020 and 2021. These comprised 78 wild and feral individuals from 12 Chinese provinces, along with 11 cultivated individuals from farmland (Figure 2; Table S3). The three varieties differ substantially in their ranges and frequency within China (Figure S1), leading to unequal sampling among them (Figure 2; Table S3). For each sampled individual, at least one herbarium specimen was made, and leaf material was collected and preserved in silica gel, with the location and habitat information (including companion species) recorded. Specimens were assigned to var. *nivea*, var. *tenacissima*, or var. *strigosa* based on the key characters described above. For ecological studies, specimens gathered from agricultural fields or greenhouses were classified as cultivated, whereas those obtained from outside cultivation but close to current agricultural activity and/or human habitation were labeled as feral. Specimens from natural forest vegetation on limestone mountains, which is the known habitat for var. *strigosa* (Zhao et al. 2024), was treated as naturally wild (henceforth “wild” for simplicity).

Herbarium specimens examined in this study were deposited in the Herbarium (KUN) of Kunming Institute of Botany, Chinese Academy of Science. In addition to the DNA sequences from individuals we collected, sequences for the *mat*K, *rbc*L, *psb*A‐*trn*H, and *trn*L‐*trn*F plastid loci were also obtained from the GenBank database. The number of additional sequences available varied from 7 (*psb*A‐*trn*H) to 22 (*trn*L‐*trn*F). Detailed species information and associated GenBank accessions are listed in Tables S3 and S4.

### 2.2 DNA Extraction, Sequencing, Assembly and Annotation

Total DNA was extracted from 0.2 g of the silica‐dried leaves using the modified 4 × CTAB method (Doyle and Doyle 1987), in which the ground leaf material were pre‐treated with a de‐sugar buffer to remove polysaccharides and cellulose. DNA quality was checked using 1% agarose gel electrophoresis and a NanoDrop ND‐1000 spectrophotometer. The DNA library (300–500 bp) was constructed using the Illumina Nextera XT DNA library construction kit. Sequencing was performed on the DNBSEQ‐T7 high‐throughput platform with a paired‐end strategy (2 × 150 bp), yielding a total data amount of around 2 Gb. The plastome assembly and annotation methods of sequences followed Li et al. (2022). The clean data obtained by high‐throughput sequencing was directly assembled using GetOrganelle v.1.7.5.3 (Jin et al. 2020), and the complete circular plastid genome was automatically output. If the circular structure could not be obtained, we visually inspected the results using Bandage v.0.8.1 (Wick et al. 2015). We then identified reliable plastid genome contigs or scaffolds by manually removing non‐target contigs from the FASTG file. The selected sequences were then manually edited and spliced to obtain a complete plastid genome. The plastid genome was annotated by Geneious v.9.0.2 (Kearse et al. 2012) using *B. nivea* var. *nipononivea* (Genbank MN189944.1) (Wang et al. 2020) as the reference and then combined with ORF (open reading frame) for correction. All coding sequences (CDS) and sequences for specific loci (*mat*K, *rbc*L, *psb*A*‐trn*H and *trn*L*‐trn*F) were extracted by Geneious v.9.0.2 based on the above annotation.

The nrDNA (nuclear ribosomal DNA, 18S‐ITS1‐5.8S‐ITS2‐26S) sequence, spanning approximately 5000–6000 bp, was similarly assembled utilizing GetOrganelle v.1.7.5.3. Following this, the resultant FASTG file and a reference sequence from *B. nivea* var. *nipononivea* (Genbank ON045579.1) were aligned and annotated using Geneious v.9.0.2. The Mafft Multiple Alignment plugins (Katoh and Standley 2013) were employed within Geneious v.9.0.2. The matrices of concatenated sequences were serialized with ActivePerl v.5.26.1 (Brown 2000). Aligned matrices were developed for the complete plastid genome, concatenated CDS, the four plastid loci, and nrDNA. All newly obtained and annotated sequences were deposited in GenBank with accession numbers (Table S3).

### 2.3 Morphological Analysis

Morphological terminology used throughout this paper follows Harris and Harris (2001). We comprehensively analyzed 11 morphological characters that vary within *B. nivea* to determine which were best for discriminating between varieties. We then selected five qualitative and six quantitative characters for statistical analysis (Kruglyak and Lander 1995; Serpico 2020). Qualitative characters comprised stigma form, leaf base, leaf tip, leaf margin, and color of the abaxial leaf blade; quantitative characters comprised leaf area, leaf length, leaf width, stipule length and width, and inflorescence length (Table S5). Stigma characters had not previously been considered for ramie infra‐specific taxonomy, but were included since floral character variation within plant species often has taxonomic significance (Garnock‐Jones and Langer 1980; Sari et al. 2016; Sari and Daryono 2018).

To assess the characters, a total of 127 specimens were examined, drawn from the 89 sampled individuals representing the three varieties (Table S3). Multiple specimens were analyzed for some individuals to capture variability. For the leaf base, tip, margin, color of the abaxial leaf blade, leaf area, leaf length, and leaf width, we chose the middle three leaves, using a Canoscan Lide220 scanner to scan and a Vernier caliper (SHAHE waterproof digital caliper), RF software transmitter, and receiver for measurement (raw data in Table S6). Leaf area was calculated using ImageJ software (Table S7). For morphological measurement of stipule characters, we selected three stipules per plant (one each from upper, middle, and lower parts) and recorded their degree of connateness; then measured their lengths and widths (Table S6). Average scores were recorded per plant for each of these characters. For the inflorescence length, similar to the stipule measurement method, we selected and measured the longest spreading inflorescence of the upper, middle, and lower stem portions (Table S6). We utilized Spearman's rank correlation coefficient (Sedgwick 2014) for qualitative characters and Pearson's correlation coefficient (Sedgwick 2012) for quantitative characters to exclude strongly correlated characters that could lead to bias in our subsequent analysis. In each comparison of two characters, if the absolute value of the coefficient is < 0.1, this implies an insignificant correlation between them.

For qualitative characters, three kinds of analysis were employed: (1) Principal Coordinates Analysis (PCoA) using the R packages “FD” (Laliberté et al. 2014), “ggfortify” (Tang et al. 2016), “ggbiplot” (Wickham et al. 2016), “ggrepel” (Slowikowski et al. 2018), “vegan” (Dixon 2003) and “ade4” (Dray and Dufour 2007); (2) plotting percentage histograms with the “ggplot2” package (Wickham et al. 2016); and (3) a chi‐squared test using the IBM SPSS Statistics 25 software (George and Mallery 2018).

For quantitative characters, we employed PCoA, boxplots (Nie et al. 2021), and pairwise comparisons. Boxplot analysis was carried out using the package “ggplot2” (Wickham et al. 2016), and IBM SPSS Statistics 25 software (George and Mallery 2018) was used for pairwise comparisons. Before pairwise comparisons, a Levene's test (Levene 1960) was conducted. If homogeneity of variances was confirmed then an LSD (Least Significant Difference) test (Williams and Abdi 2010) was used; if not confirmed, a Tamhane's T2 test (Tamhane 1979) was used.

To further assess the utility of qualitative and quantitative characters for discrimination between *B. nivea* varieties, random forest analysis was carried out using the “randomForest” package (Strobl et al. 2007).

### 2.4 Phylogenetic Analysis

According to the phylogenetic framework of Urticaceae (Wu et al. 2013; Wu et al. 2018), *Astrothalmus* C.B.Rob. and *Debrageasia* Gaudich. are the genera most closely related to *Boehmeria*. We therefore selected two species of *Debregeasia*, one species of *Astrothalmus*, and seven other species of *Boehmeria* as outgroups (Table S3). All sequences have been uploaded to NCBI (National Center for Biotechnology Information).

We constructed maximum likelihood (ML) and Bayesian inference (BI) trees based on each of four datasets: (a) complete plastomes, comprising the Large Single Copy (LSC), Small Single Copy (SSC), Inverted Repeat A (IRa) and Inverted Repeat B (IRb) regions, (b) all CDS sequences concatenated, (c) nrDNA sequences, and (d) complete plastomes + nrDNA sequences.

To analyze the phylogeny of ramie with a larger sample set, we also analyzed the four plastid loci *mat*K, *rbc*L, *psb*A‐*trn*H, and *trn*L‐*trn*F using sequences previously reported in the Genbank database (Table S4), together with the sequences extracted from our own complete plastid sequences. We constructed phylogenetic trees using individual and concatenated sequences from the loci. This approach also allowed us to compare the phylogenetic relationships inferred from individual loci and from complete plastomes.

Maximum Likelihood (ML) trees were constructed using RAxML v.8.2.11 (Stamatakis 2014) with 1000 rapid bootstrap replicates. For Bayesian Inference (BI) with MrBayes v.3.2.7 (Ronquist et al. 2012), runs of 1,000,000 generations were performed. We checked the potential scale reduction factor (PSRF) values for convergence, and all datasets showed PSRF values close to 1.0, indicating convergence. For each analysis, the best‐fit substitution model was selected using jModeltest v.2.1.7 (Posada 2008) with the Akaike Information Criterion (AIC). Phylogenetic trees were then visualized in iTOL (Letunic and Bork 2021). We also used the R package “phytools” (Revell 2012) to compare the topologies between the two trees.

### 2.5 Ecological Niche Modeling

We carried out ecological niche modeling (ENM) of the three *B* . *nivea* varieties using Maxent v.3.4.3 software and species occurrence data from our fieldwork (see Table S1). Environmental data layers for 19 bioclimatic variables were obtained from the WORLDCLIM database (http://www.worldclim.org) at a spatial resolution of 2.5 arc minutes. The combined data of occurrence and bioclimatic factors were projected across different time periods as follows: (1) var. *nivea* and var. *tenacissima*, projections were made for the Present (1970–2000) and Future (ad 2100). Historical reconstructions for these varieties were not attempted, as we could not predict the distribution of potential wild ancestors outside the cultivated areas (or former cultivated areas) because buffer zones used in the analysis are based on existing distribution data that does not include known wild types. (2) Data for the wild variety, var. *strigosa*, were projected across four periods: Last Interglacial (LIG, 116–129 ka BP), Last Glacial Maximum (LGM, 18–26.5 ka BP), Present, and Future. To avoid overfitting, ArcGIS v.10.8 software (http://desktop.arcgis.com/zh‐cn) was used to remove strongly correlated environmental factors. The receiver operating characteristic (ROC) curve and area under the curve (Salmaki et al. 2013) were used to evaluate the performance of the well‐established model (Swets 1988; Vanagas 2004). Finally, ArcMap in ArcGIS v.10.8 software was used to draw the distributions of suitable environments at different times.

## 3 Results

### 3.1 Morphological Characters Analysis

**FIGURE 3:**
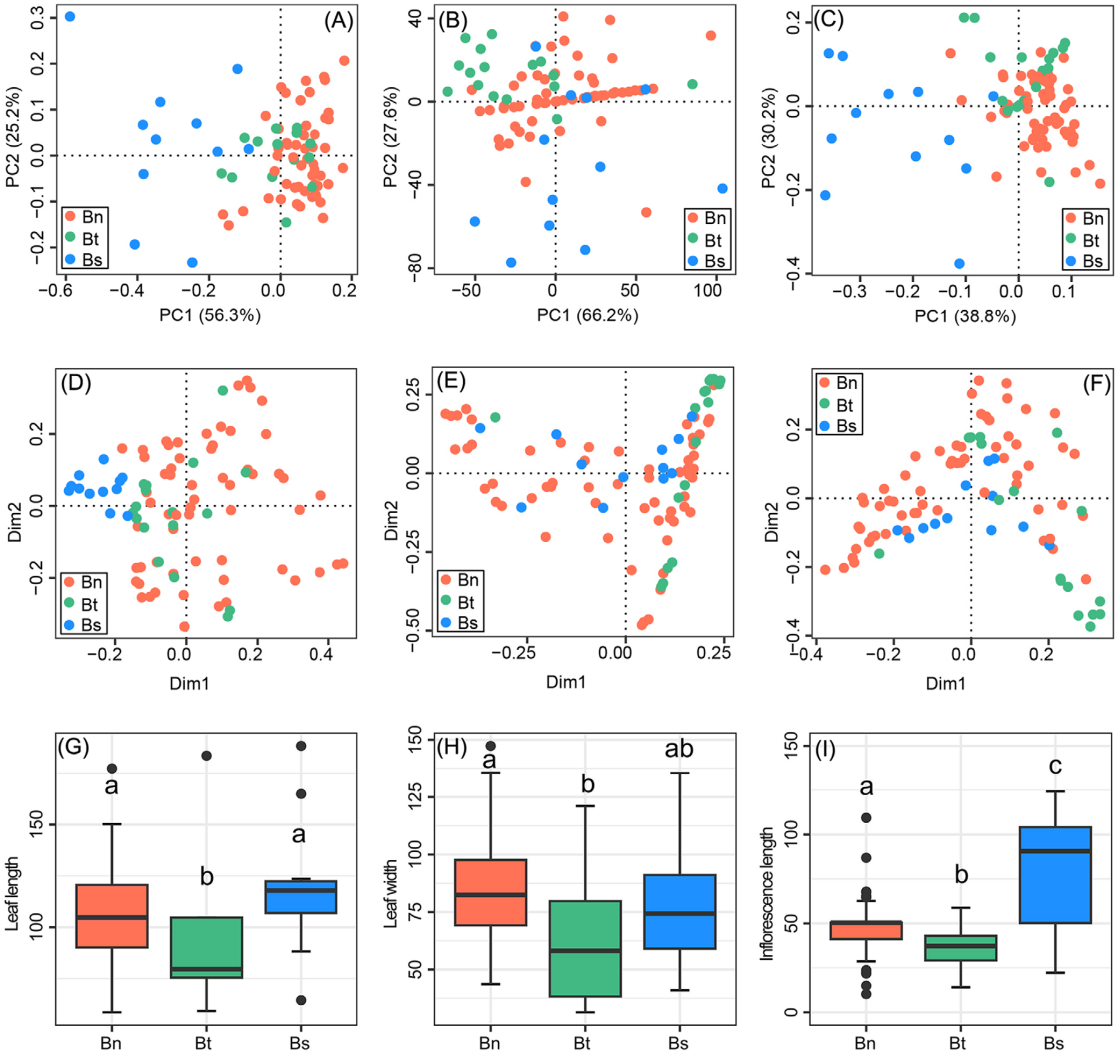
Differences in morphological characteristics among *Boehmeria nivea*. Bn, Bt, and Bs represent var. *nivea*, var. *tenacissima*, and var. *strigosa*, respectively. (A–C) PCoA results for morphological characters: (A) qualitative characters; (B) quantitative characters; (C) all characters. (D–F) random forest analysis of morphological characters: (D) qualitative characters; (E) quantitative characters; (F) all characters. (G–I) boxplots of quantitative characters: different letters (a, b, c) indicate significant differences, whereas varieties sharing a letter are not significantly different. (G) leaf length; (H) leaf width; (I) inflorescence length.

Although stigma form is often used as a diagnostic character at the genus level within Urticaceae (Wang and Chen 1995; Chen et al. 2003; Wu et al. 2015), no differences in this trait were observed among the three varieties. The Spearman's rank correlation coefficient analysis indicated no significant correlations among the other four qualitative characters examined, that is, leaf base, leaf tip, leaf margin, and color of the abaxial leaf blade (Table S8). Pearson's correlation coefficient showed that three of the six quantitative characters were uncorrelated with one another: leaf length, leaf width, and inflorescence length (Table S9). Consequently, these seven characters were retained for further analysis. Principle Components Analysis (PCoA) of the four qualitative characters revealed minimal overlap between var. *strigosa* and the other two varieties, while var. *nivea* and var. *tenacissima* clustered together (Figure 3A). When only the three quantitative characters were analyzed, there was overlap between all three varieties (Figure 3B). Additionally, when all qualitative and quantitative characters were analyzed together, the results were similar (Figure 3C). Thus, PCoA analysis could distinguish var. *strigosa* from the other two varieties based on qualitative characters, and on all characters, but not on quantitative characters alone.

The random forest algorithm scatter plot results (Figure 3D–3F) were consistent with PCoA (Figure 3A–3C). Based on the random forest model, the character that contributed most to distinguishing the three varieties was color of the abaxial leaf blade, followed by inflorescence length (Table 1). The clearest discrimination between var. *nivea* and var. *strigosa* was provided by color of the abaxial leaf blade. For discrimination between var. *tenacissima* and var. *strigosa*, the clearest discrimination was provided by leaf width (Table 1).

**TABLE 1:** Summary of random forest algorithm contribution percentages based on all characters of *Boehmeria nivea* .

Characters	*B. nivea* var. *nivea* ^a^	*B. nivea* var. *tenacissima* ^a^	*B. nivea* var. *strigosa* ^a^	Mean Decrease in model Accuracy^b^	Mean Decrease in Gini value^c^
Leaf base	4.08	6.01	1.3	6.69	3.59
Leaf tip	2.8	8.96	−2.11	6.66	2.93
Leaf margin	8.81	4.09	13.76	14.26	6.05
Color of the abaxial leaf blade	**20.9**	7.55	**26.97**	**27.52**	**11.45**
Leaf length	4.95	11.34	3.48	11.4	5.56
Leaf width	9.23	**12.81**	−2.17	13.97	6.52
Inflorescence length	7.28	12.35	8.93	15.15	7.15

^a^ The numbers in bold indicate the maximum contribution rate value within each column.

^b^ “Mean Decrease in model Accuracy” represents the value by which the accuracy of the model decreases after randomly disrupting the character in that row. The size of this value is proportional to the importance of that character in distinguishing taxa (Breiman 2001; Liaw and Wiener 2002; Strobl et al. 2007). The highest values are in bold.

^c^ “Gini” refers to the statistical measure of inequality used to evaluate feature importance in classification models (Breiman 2001). Values here represent the decrease in Gini value for the model after randomly disrupting the character in that row. The size of this value is proportional to the importance of that character in distinguishing taxa, and the highest values are in bold.

Percentage histograms of the four retained qualitative characters indicated a significant difference between varieties in the proportion of individuals exhibiting more than one state for one character (Figure 1G; Figure S2D). This was consistent with the chi‐square test results, which assessed the ability of each character to distinguish the three varieties, with *p* < 0.05 for all four qualitative characters (Table S10). PCoA, percentage histograms, and chi‐squared test results together showed that these four qualitative characters could distinguish the three varieties.

Conversely, boxplot analysis of the three quantitative characters showed overlap between varieties (Figure 3G–3I), again indicating these characters have weak discrimination power. However, while overall discrimination by the quantitative characters is limited, significant differences among the varieties can still be observed in specific statistical analysis (Table S11). Levene's test revealed that leaf length exhibited variance homogeneity among the three varieties, and the LSD result revealed significant differences between var. *tenacissima* and the other two varieties. Leaf width also exhibited variance homogeneity, and LSD results indicated significant differences between var. *nivea* and var. *tenacissima*. For inflorescence length, there was no homogeneity of variance, but Tamhane's T2 test demonstrated a significant difference among the three varieties (Table S11).

### 3.2 Phylogenetic Relationships Within *B. nivea*

Consistent ML and BI topologies were produced by analyses of complete plastomes (Figure 4) and some data subsets or combinations (Figures S3–S8 and S11–S14). With the *rbc*L and “*mat*K *+ rbc*L *+ psb*A*‐trn*H *+ trn*L*‐trn*F” plastid subsets, ML and BI topologies were discrepant (Figures S9, S10, S15, and S16).

**FIGURE 4:**
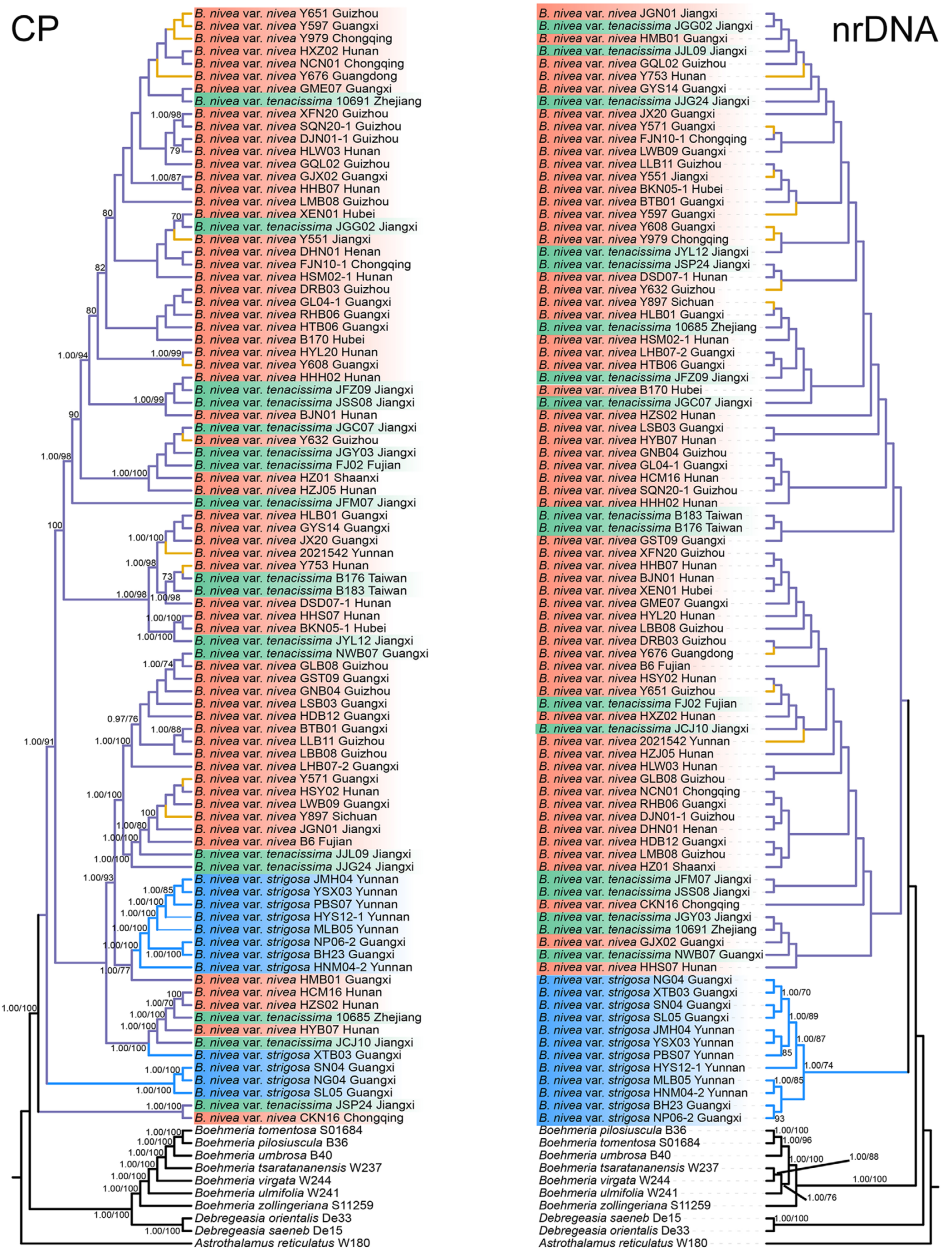
Complete plastid genome (CP, left) and nuclear ribosomal DNA (nrDNA, right) trees for *B. nivea* and related species: consensus phylogenetic trees constructed using Maximum Likelihood (ML) and Bayesian Inference (BI) methods. Numbers at nodes indicate support values, BI posterior probabilities (PP) at left, ML bootstrap values at right, with only values ≥ 0.95 or ≥ 70% respectively shown. Blue, purple and yellow lines represent wild, feral and domesticated accessions, respectively. Each tip is labeled with botanical name, sample ID, and province (outgroups excepted).

Grouping all individuals from the three varieties as a monophyletic group (i.e., *B. nivea* ), separate from all *Boehmeria* species in the outgroups, was supported by all datasets (Figure 4; Figures S3–S10 and S13–S16) except for *psb*A‐*trn*H (Figures S11 and S12). This grouping was weakly supported by the nrDNA data (PP < 0.95, BS < 70%) and strongly supported by the complete plastome, CDS, “complete plastome + nrDNA” and “*mat*K + *rbc*L + *psb*A‐*trn*H + *trn*L‐*trn*F” datasets (Figure 4; Figures S3–S6).

Within the nrDNA tree, var. *strigosa* formed a clear monophyletic group, with a posterior probability (PP) of 1.0 and a bootstrap value (BS) of 74%, but was biphyletic with other datasets (Figure 4; Figures S3–S16). The other two varieties together formed a weakly supported (bootstrap value < 70%) monophyletic nrDNA clade.

Only the nrDNA dataset divided *B. nivea* into two sister clades. With the other eight datasets based wholly or mostly on plastome data, the three varieties formed nested topologies (Figure 4; Figures S3–S16). The topologies produced by complete plastomes, CDS, and complete plastomes + nrDNA were very similar (Figure 4; Figures S3–S6). With these three datasets, all individuals of var. *strigosa*, except XTB03, formed two distinct clades that branched off very early. The “*mat*K *+ rbc*L *+ psb*A*‐trn*H *+ trn*L*‐trn*F” phylogeny also resolved these two clades, but with different branching orders (Figures S15 and S16). When the single loci of *mat*K, *psb*A*‐trn*H, and *trn*L*‐trn*F were analyzed, var. *strigosa* individuals did not cluster into monophyletic groups (Figures S7–S8 and S11–S14). With the *rbcL* locus, all but four individuals or var. *strigosa* formed a single clade (Figures S9 and S10).

Individuals of the three varieties from each province did not cluster into clearly separate, geographically defined clades (Figure 4; Figures S3–S16), and individuals from cultivated, feral, and wild habitats did not form separate clades defined by habitat (Figure 4; Figures S3–S16).

### 3.3 Niche Differentiation

**FIGURE 5:**
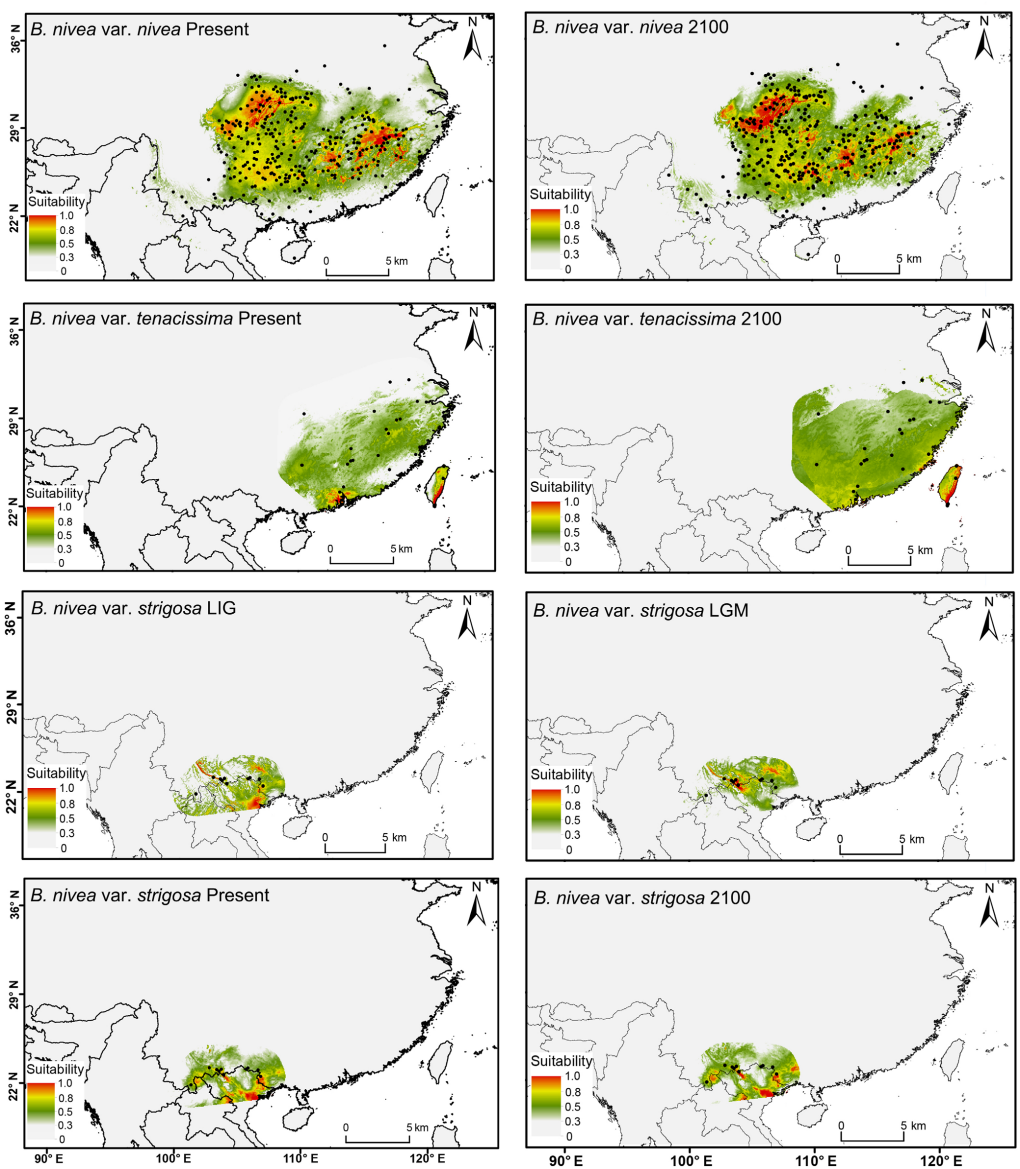
Potential distributions of three *Boehmeria nivea* varieties in different periods: LIG (Last Interglacial), LGM (Last Glacial Maximum), Present (1970–2000), and Future (ad 2100) (as defined in text). The color gradient from green to red indicates low to high plant fitness (suitability for a given area).

The AUC values of our species distribution model simulations for different time periods ranged from 0.789 to 0.876 (Figure S17), indicating “good” performance of the model simulations. Among the environmental variables selected for niche simulation (Table S12), precipitation emerged as the predominant environmental factor shaping the potential distribution of var. *nivea* in different periods, and var. *strigosa* in the Future (ad 2100) period. In contrast, for var. *tenacissima* in the Present and Future periods, and for var. *strigose* in past (LGM, LIG) and Present periods, temperature was the main factor influencing potential distribution (Table S13). In different periods, var. *nivea* and var. *tenacissima* exhibited a significant overlap in their predicted distributions in China (Figure 5), while var. *strigosa* showed no overlap with the distributions of var. *tenacissima* in China during any period, and very limited overlap with var. *nivea* (Figure 5).

## 4 Discussion

### 4.1 Subdivision of *B. nivea* (Ramie) Based on Morphological Evidence

The qualitative characters of leaf base, leaf tip, leaf margin, and color of the abaxial leaf blade could significantly distinguish the three ramie varieties based on random forest analysis (Table 1) and chi‐squared test (Table S10). Color of the abaxial leaf blade showed significant discrimination among the three varieties, as was evident in random forest analysis (Table 1). A chi‐squared test indicated that each of the four qualitative characters alone could distinguish the three varieties (Table S10). Conversely, quantitative characters were found to have little discriminatory power between the three varieties (Figure 3B, Figure 3E; Figure 3G‐I; Table S11). Whereas qualitative characters are predominantly governed by genetic information, quantitative characters are often more influenced by environmental factors (Mudge 1909; Falconer 1996). However, the relationship between these characters is more complex, as qualitative characters can also be influenced by environmental conditions, and quantitative characters are significantly under genetic control.

At present, *B. nivea* var. *nivea* is distributed throughout the middle and lower reaches of the Yangtze River, var. *tenacissima* mainly in southeastern China, and var. *strigosa* in southeastern Yunnan and southwestern Guangxi. There is currently no geographical overlap between var. *tenacissima* and var. *strigosa*, while both have significant overlap with var. *nivea* (Figure 2; Figure S1). Var. *strigosa* comprises only naturally wild material, whereas var. *nivea* and var. *tenacissima* each contain a mix of cultivated and feralized material, with var. *nivea* mostly cultivated and var. *tenacissima* usually feralized. For var. *nivea* and var. *tenacissima*, no populations are currently considered (or known) to be naturally wild.

### 4.2 Subdivision of *B. nivea* (Ramie) Based on Phylogeny and Ecology

Phylogenetic analyses using ML and BI methods could not reliably or completely separate the three varieties. All examined individuals of var. *strigosa* except for accession XTB03 were resolved into two well‐supported monophyletic clades towards the base of the complete plastomes phylogeny (Figure 4). Conversely, the nrDNA phylogenetic trees resolved var. *strigosa* as monophyletic with relatively strong support (Figure 4). The other two varieties were intermixed in both analyses (Figure 4). Although informative, the nrDNA regions sequenced comprise only 5000–6000 bp and represent just a small fraction of the nuclear genome. A true understanding of relationships among these taxa will require much larger nuclear sampling and analysis of multiple gene trees (Kipkoech et al. 2024) or whole‐genome resequencing (Ma et al. 2018).

Both PCoA analysis (Figure 3A–C) and the random forest analysis (Figure 3D–F) indicated that var. *strigosa* was a morphologically distinct variety, and the consistency between these two methods greatly enhanceed the support for this and is consistent with the distinct position of var. *strigosa* indicated by nrDNA analysis. Although var. *nivea* and var. *tenacissima* can be clearly distinguished from one another by morphology, they did not cluster separately in the phylogenetic trees. This may relate to var. *nivea* being a domesticated taxon, as certain homoplasies traits could have been repeatedly acquired during parallel domestication events. Similarly, domestication traits might have been repeatedly lost during multiple parallel derivations of var. *tenacissima* from within var. *nivea* via feralization. Furthermore, hybridization and introgression frequently occur among closely related species and infraspecific taxa (Goulet et al. 2017), and might be indicated by noncongruence in the topologies of the plastid and nrDNA trees shown in (Figure 4, most obviously (but not limited to) the branches containing var. *strigosa*.

Currently, geographic separation limits the potential for hybridization and potential introgression in *B. nivea* (Figure 2; Figure S1); however, there may have been much greater overlap in the past, when cultivation was more common and widespread, along with a wider distribution of feralized populations derived from cultivations. A complex history of gene flow between varieties may be why the phylogeny based on complete plastomes does not resolve any of the three *B. nivea* varieties as monophyletic. Thus, multiple lines of evidence are needed to generate a functional infraspecific classification.

The potential distribution of the three varieties (Figure 5) demonstrated that var. *strigosa* is ecologically distinct, as well as genetically and morphologically distinct. In addition, var. *strigosa* occurs only on limestone mountains, providing further ecological separation not covered by environmental factors included in the present ecological niche models.

### 4.3 Infraspecific Divisions in a Species That Contains Cultivated, Feral, and Naturally Wild Material

Feralization can be regarded as a natural process following deliberate anthropogenic alteration, and it is highly likely to involve multiple parallel events, especially where cultivation has rapidly decreased, leaving disturbed habitats open for colonization (Gressel 2005). The process will likely involve the rapid loss of specific character traits that had arisen and been maintained by strong artificial selection, meaning that feral populations derived independently of one another may differ from cultivated material in the same characters (Ellstrand et al. 1999). Hence, the pattern observed here of intermixture between cultivated and feral material is to be expected even in the arguably unlikely absence of subsequent crop‐to‐feral and/or feral‐to‐crop gene flow (Reagon et al. 2010). This creates significant challenges for the taxonomist, which the current study has attempted to address using an integrative approach.

As expected from the above reasoning, we found that *B. nivea* var. *tenacissima*, which predominantly comprises feral material, is morphologically but not genetically distinct. Feral material cannot be expected to be monophyletic, and recognition at the varietal level must instead be supported by morphology and ecology (Hufford and Mazer 2003). Gene flow between cultivated and feral varieties may make little difference, as their distinctness can be maintained by artificial and natural selection, respectively (Ellstrand and Schierenbeck 2000). Indeed, there might be parallels here with Wu's (2001) genic species concept, with only small parts of the genome separating cultivated and feral forms, while the rest of the genome moves freely between them.

Given the complex and close genetic relationship between *B. nivea* var. *nivea* and var. *tenacissima*, the absence of clear ecological separation between them in terms of climate preference (Figure 5) is unsurprising. Additionally, it is worth considering whether *B. nivea* has naturalized in regions such as northern China, the Korean peninsula, and Japan, as this ecological shift could define the outer boundaries of the species' possible natural range. Nor is it clear whether they will diverge over time because the diverging influence of artificial versus natural selection might be balanced by the homogenizing effect of gene flow between them and repeated feralization events.

We did not find clear evidence for gene flow between the feral var. *tenacissima* and naturally wild var. *strigosa*, possibly because of their past and present geographical separation (Figure 5; Figure S1). The non‐monophyly of var. *strigosa* has two possible explanations: (1) cultivated material and feral populations are ultimately derived from diversity present within ancestral populations of var. *strigosa*, rendering the latter paraphyletic, and (2) the var. *strigosa*/var. *tenacissima* group is derived from an unknown ancestral, naturally wild population that hybridized with var. *strigosa*. In the absence of known naturally wild populations of the var. *strigosa*/var. *tenacissima* group, we consider the second possibility unlikely. The observation that species cannot always be expected to be monophyletic (Rieseberg and Brouillet 1994) indicates that infraspecific taxa may also be non‐monophyletic; however, this does not render them biologically meaningless. A comprehensive study of morphology, genetics, and ecology is needed and allows for a holistic assessment of the biological significance of a proposed variety (Van de Wouw et al. 2010).

Infraspecific taxa are commonly first recognized through morphological observation and later tested with DNA evidence where possible. In this study, molecular evidence revealed that var. *nivea* and var. *tenacissima* cannot be wholly distinguished at this stage. The combined analysis of morphology, phylogeny, and ecology highlighted the uniqueness of var. *strigosa*. In summary, integrating multiple analytical approaches has increased our understanding of the differentiation present within *B. nivea* today and the likely underlying processes leading to this differentiation.

PCoA and random forest analyses of morphological characters failed to significantly differentiate between var. *nivea* and var. *tenacissima* (Figure 3), showing significant overlap in their morphology. Molecular data indicated a deep‐level nesting relationship between them, and they could not be clearly distinguished in phylogenetic analyses of any dataset examined here (Figure 4; Figures S3–S16). The two not only have a broad observed geographical overlap (Figure S1) but also exhibit very similar likely distributions based on ecological niche modeling (Figure 5).

Although chi‐square tests of qualitative characters can differentiate var. *tenacissima* from other varieties (Table S10), this differentiation is limited to characters related to the leaves. This might be because var. *nivea* mainly includes cultivated forms suitable for living environments with better conditions for leaf growth, var. *tenacissima* primarily occurs in less suitable wild environments. The significant differences in leaf characters between the two may reflect differences in their ecological adaptability, but other factors, such as stochastic genetic differences due to founder effects, should also be considered.

Considering all the above analyses, we believe that var. *tenacissima* should be regarded as a synonym of var. *nivea*. As already noted, there is clear ecological and morphological separation between these varieties and var. *strigosa*, which only occurs as naturally wild populations.

## 5 Conclusions

Feralization can generate a complex pattern of variation within a species, where morphological characters may not correspond to genetic relatedness. However, integrative taxonomy allows the subdivision of feralized species to be better understood. Here, we examined the naturally wild, cultivated, and feral material of the economically important ramie ( *Boehmeria nivea* ), using morphological, genetic, and ecological data. Our results confirmed that the recently characterized var. *strigosa*, comprising naturally wild material, is biologically meaningful, and indicated that this is the closest natural relative of cultivated and feralized ramie.

The results also lead us to recommend that var. *tenacissima* be synonymized with var. *nivea* because the two are phylogenetically inseparable. In our view, *B. nivea* is comprised of only two varieties, var. *strigosa* (a wild type) and var. *nivea* (a domesticated form, including derived wild populations, and lacking a known wild progenitor). Substantial genetic admixture between cultivated and feralized material might be due to repeated parallel derivations of the latter via independent feralization events and/or gene flow between the varieties recognized until now. Further research is necessary with greatly increased nuclear genome coverage to strengthen support for var. *strigosa* as a monophyletic group and to understand the discordance between morphological classification and plastid phylogenetic trees.

Overall, this research provides a new empirical foundation and theoretical basis for understanding the present diversity of wild and cultivated forms of ramie, the taxonomic significance of previously recognized ramie varieties, and the biological processes that have produced diversity within this species. In this work, we have emphasized the possible role of feralization in generating infraspecific variation, although we cannot yet identify the genetic origins of cultivated ramie, which may have been diverse already when cultivation began. There is still much to learn regarding the distribution and diversity of wild ramie populations, including those recognized as var. *strigosa*. This leads to an important consideration for conservation purposes and ecosystem research: to safeguard the potential future development and utilization of ramie as a crop, wild populations should be protected where possible for their research value and their potential value as resources for plant breeding in the future. In addition, the study of wild and cultivated populations outside China is clearly needed to give a more complete picture of the evolution of *B. nivea* and its history as a cultigen.

## Author Contributions

**Ying Zhao:** formal analysis (lead), validation (equal), visualization (equal), writing – original draft (lead). **Richard I. Milne:** writing – review and editing (lead). **Jie Liu:** project administration (lead). **Zhi‐Peng Li:** software (equal), writing – review and editing (equal). **Xiao‐Gang Fu:** supervision (equal), writing – review and editing (equal). **Ke Li:** investigation (equal). **Amos Kipkoech:** writing – review and editing (lead). **Zeng‐Yuan Wu:** project administration (lead), writing – review and editing (lead).

## Conflicts of Interest

The authors declare no conflicts of interest.

## Supporting information


Tables S1‐S13.

Figures S1‐S17.

## Data Availability

The sequences presented in this study can be accessed at NCBI GenBank (https://www.ncbi.nlm.nih.gov/nucleotide/); the list of accession numbers can be found in Tables S3 and S4.
