# Supplementary material for: An Integrated Study of Ramie (Boehmeria nivea), and Its Wild, Cultivated, and Feral Forms
Source: Ecol Evol. 2025 Mar 20;15(3):e71126. doi: 10.1002/ece3.71126 (PMC11925647; doi:10.1002/ece3.71126)
Supplement: Supplementary file 2 — Figures S1‐S17. [file ECE3-15-e71126-s002.pdf]

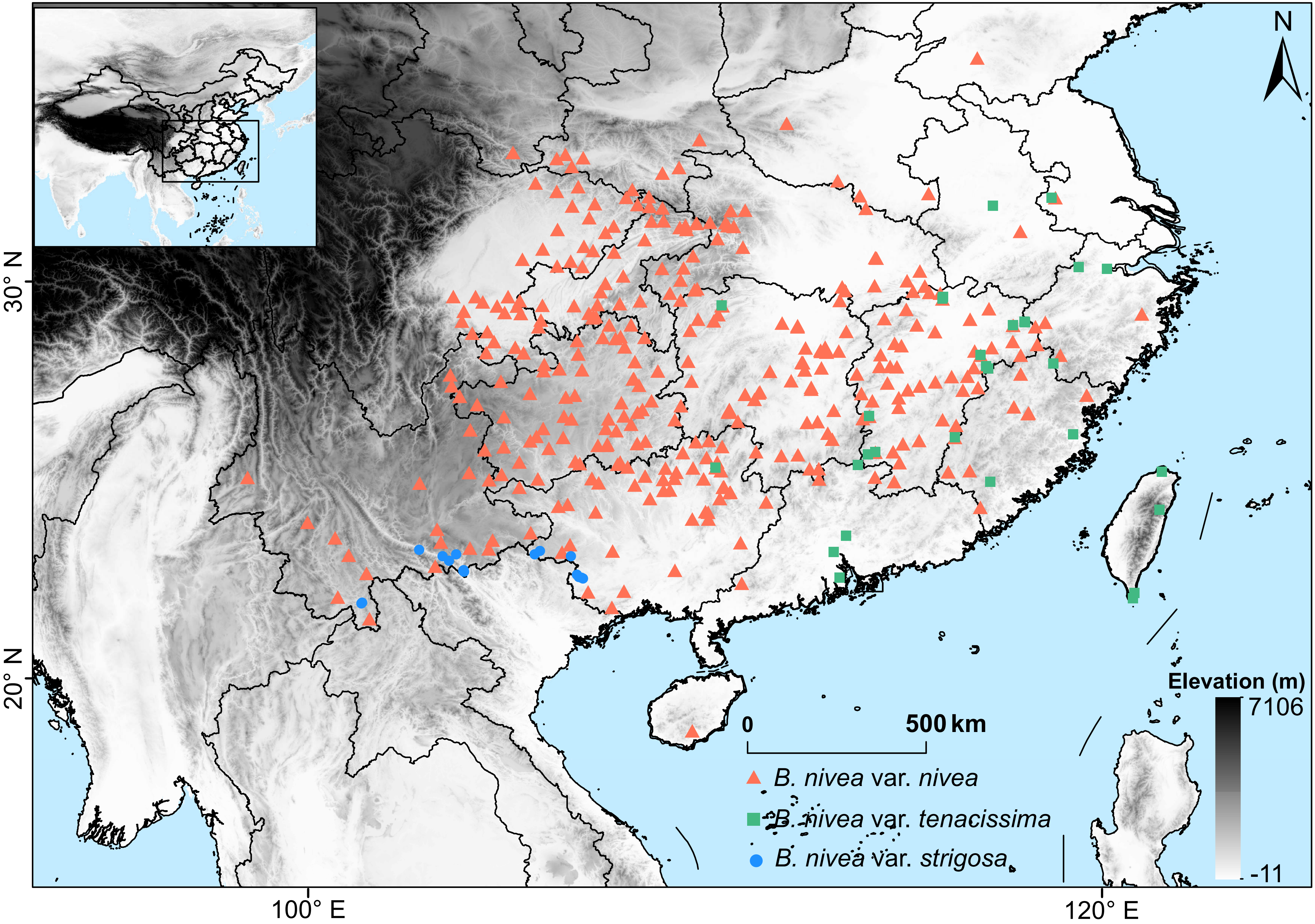

Figure S1. Distribution map of *Boehmeria nivea* in China (based on unpublished data from the field work and examination of herbarium specimens by the authors and Wu Zeng-Yuan's research group).

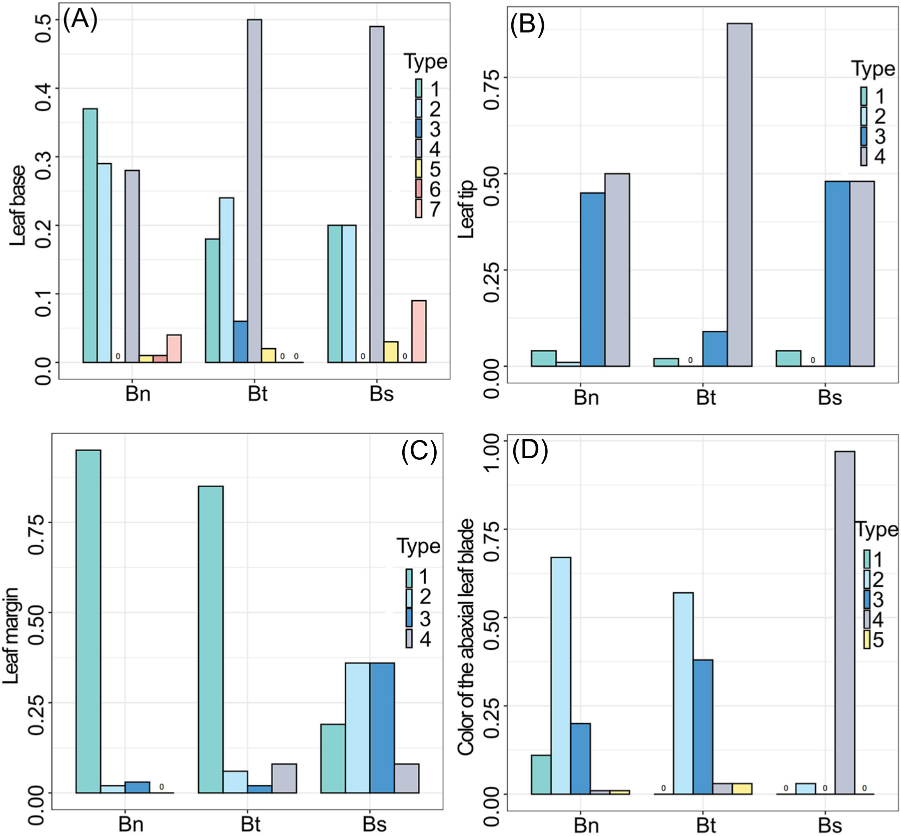

Figure S2. Percentage histograms of qualitative traits.

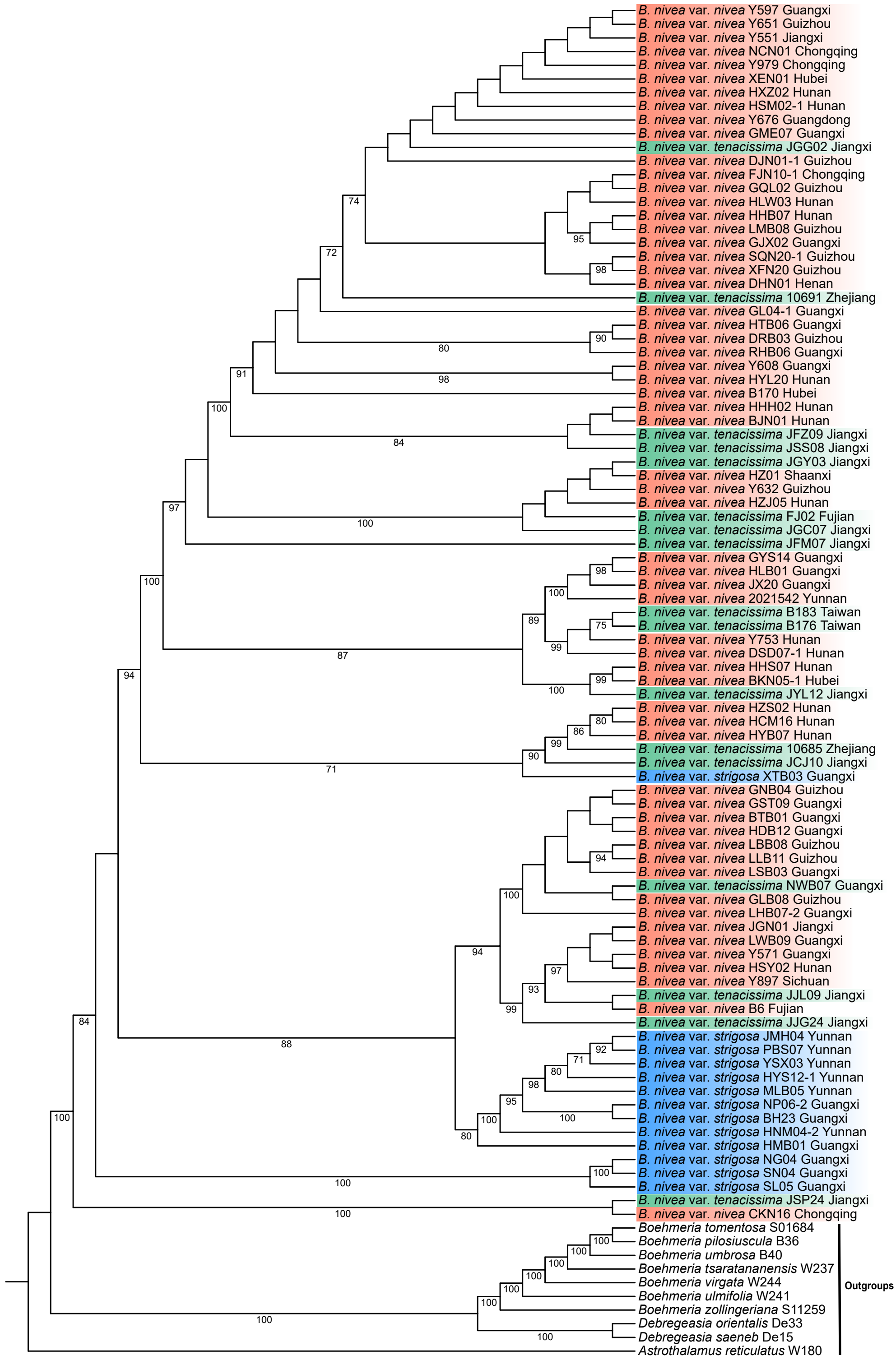

Figure S3. Maximum likelihood phylogenetic tree of *Boehmeria nivea* constructed using CDS dataset (support values only shows ≥ 70%). Each node consists of variety name_sample ID_ Province except outgroups.

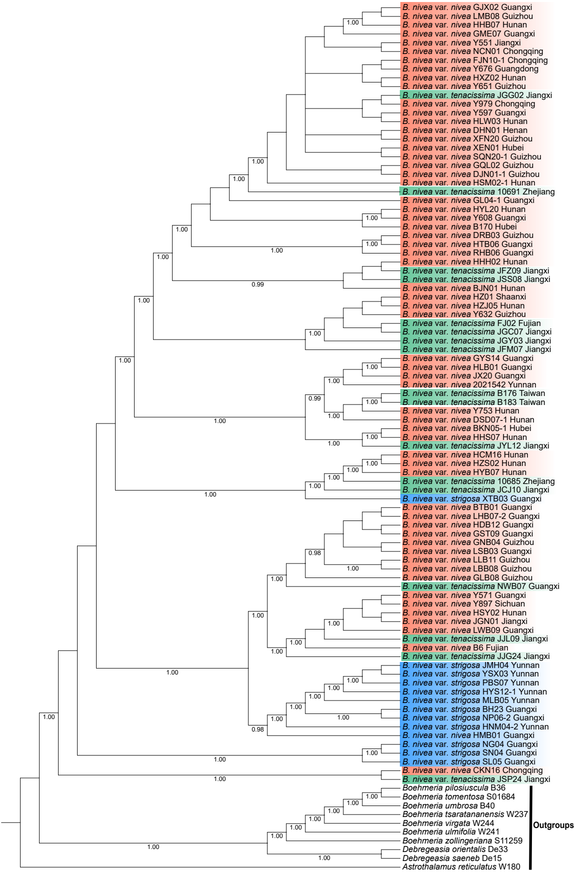

Figure S4. Bayesian phylogenetic tree of *Boehmeria nivea* constructed using CDS dataset (support values only shows ≥ 0.95). Each node consists of variety name_sample ID_Province except outgroups.

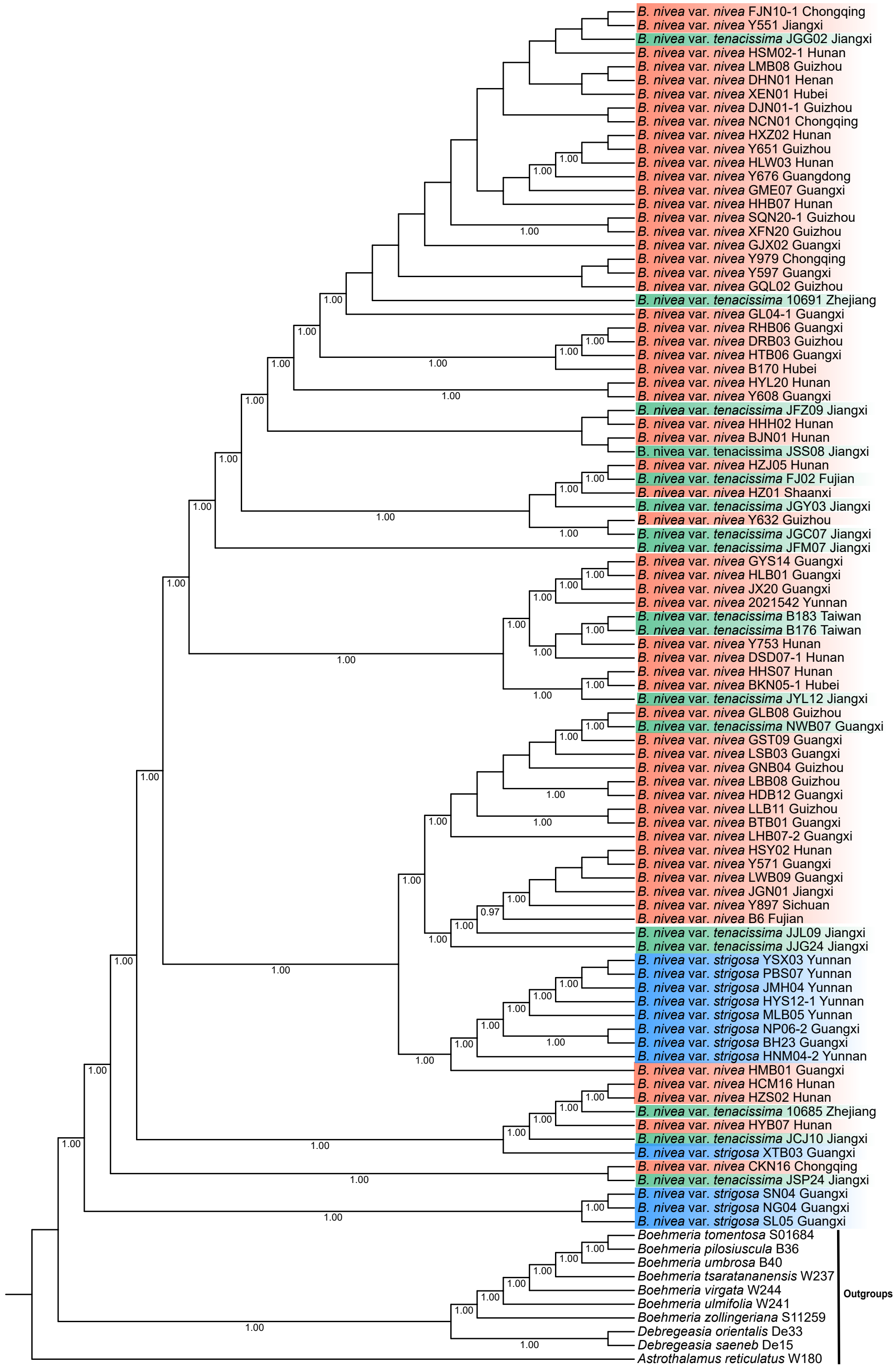

Figure S5. Bayesian phylogenetic tree of *Boehmeria nivea* constructed using complete plastomes + nrDNA dataset (support values only shows ≥ 0.95). Each node consists of variety name_sample ID_Province except outgroups.

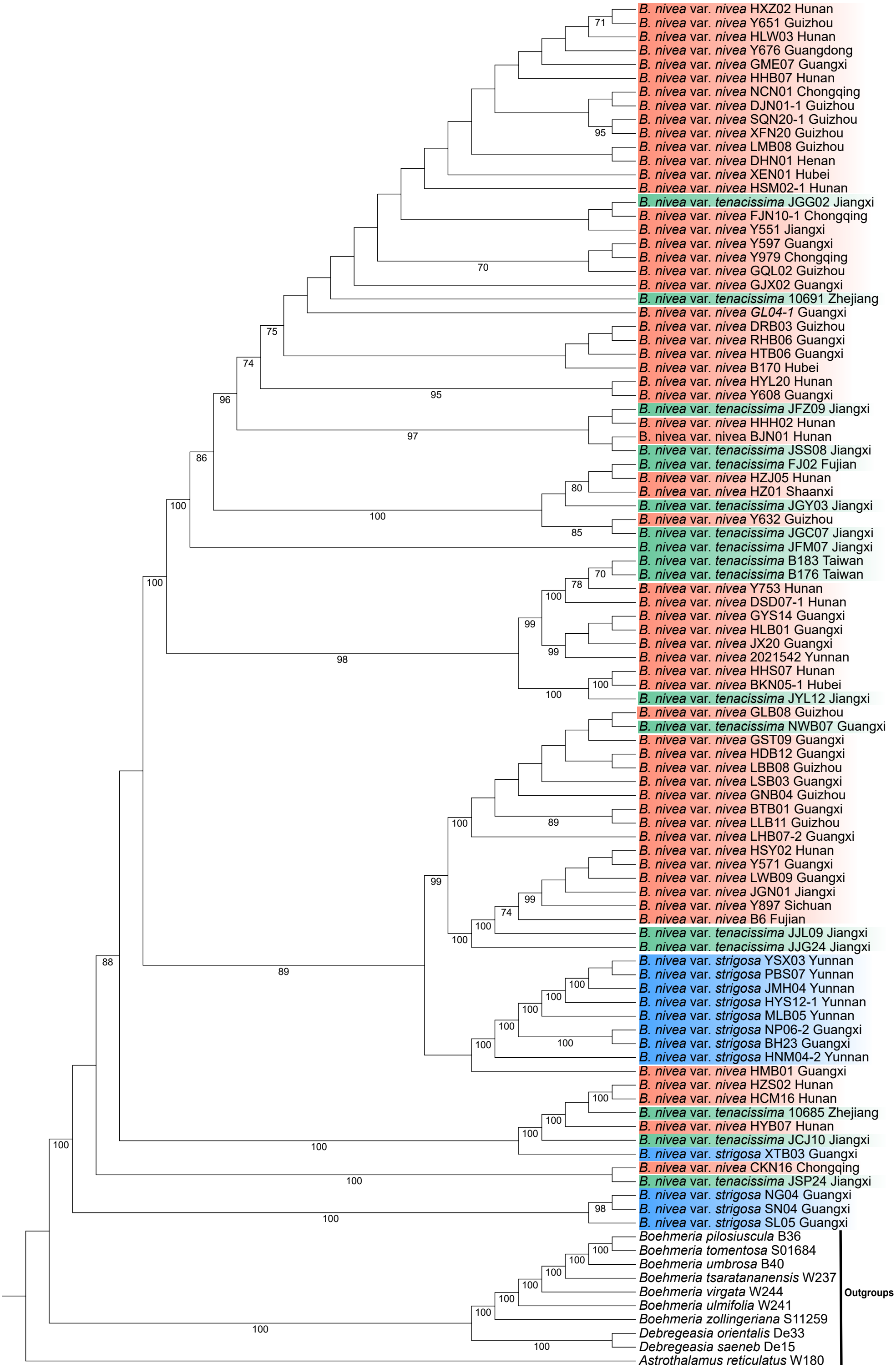

Figure S6. Maximum likelihood phylogenetic tree of *Boehmeria nivea* constructed using complete plastomes + nrDNA dataset (support values only shows ≥ 70%). Each node consists of variety name_sample ID_Province except outgroups.

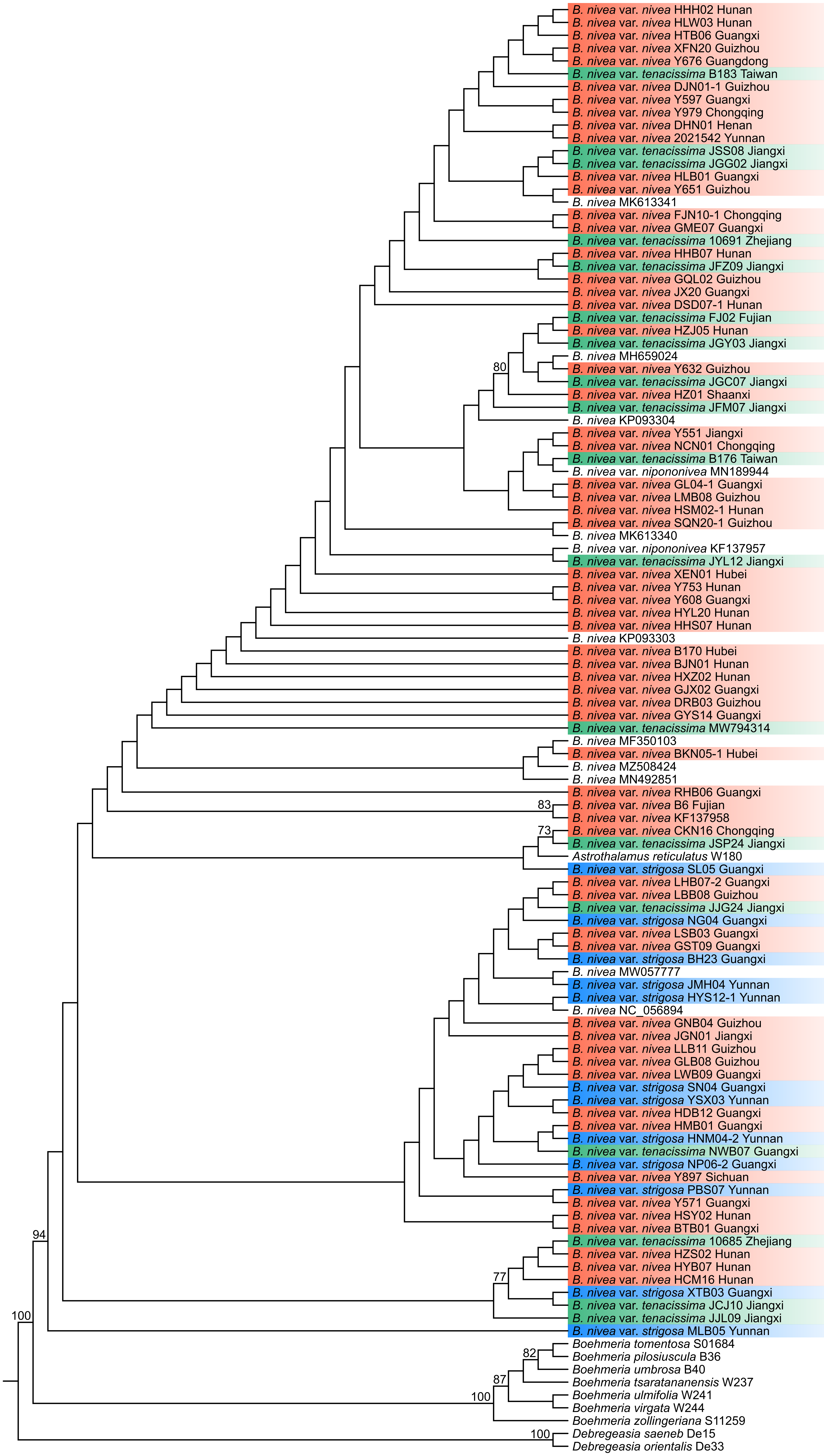

Figure S7. Maximum likelihood phylogenetic tree of *Boehmeria nivea* constructed using *mat*K dataset (support values only shows ≥ 70%). Each node consists of species name_sample ID_Province except outgroups and downloaded sequences from NCBI.

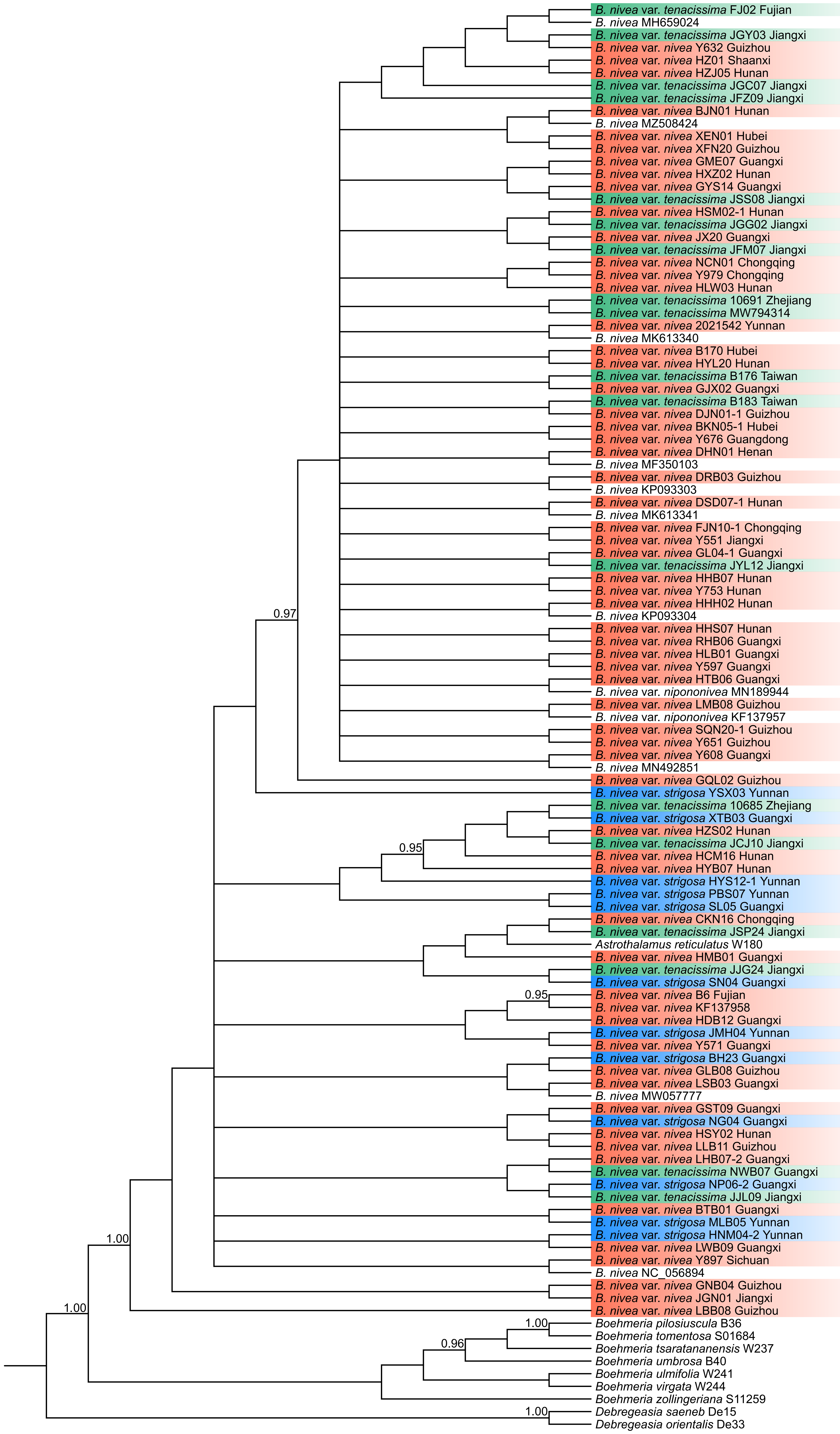

Figure S8. Bayesian phylogenetic tree of *Boehmeria nivea* constructed using *mat*K dataset (support values only shows ≥ 0.95). Each node consists of species name_sample ID_Province except outgroups and downloaded sequences from NCBI.

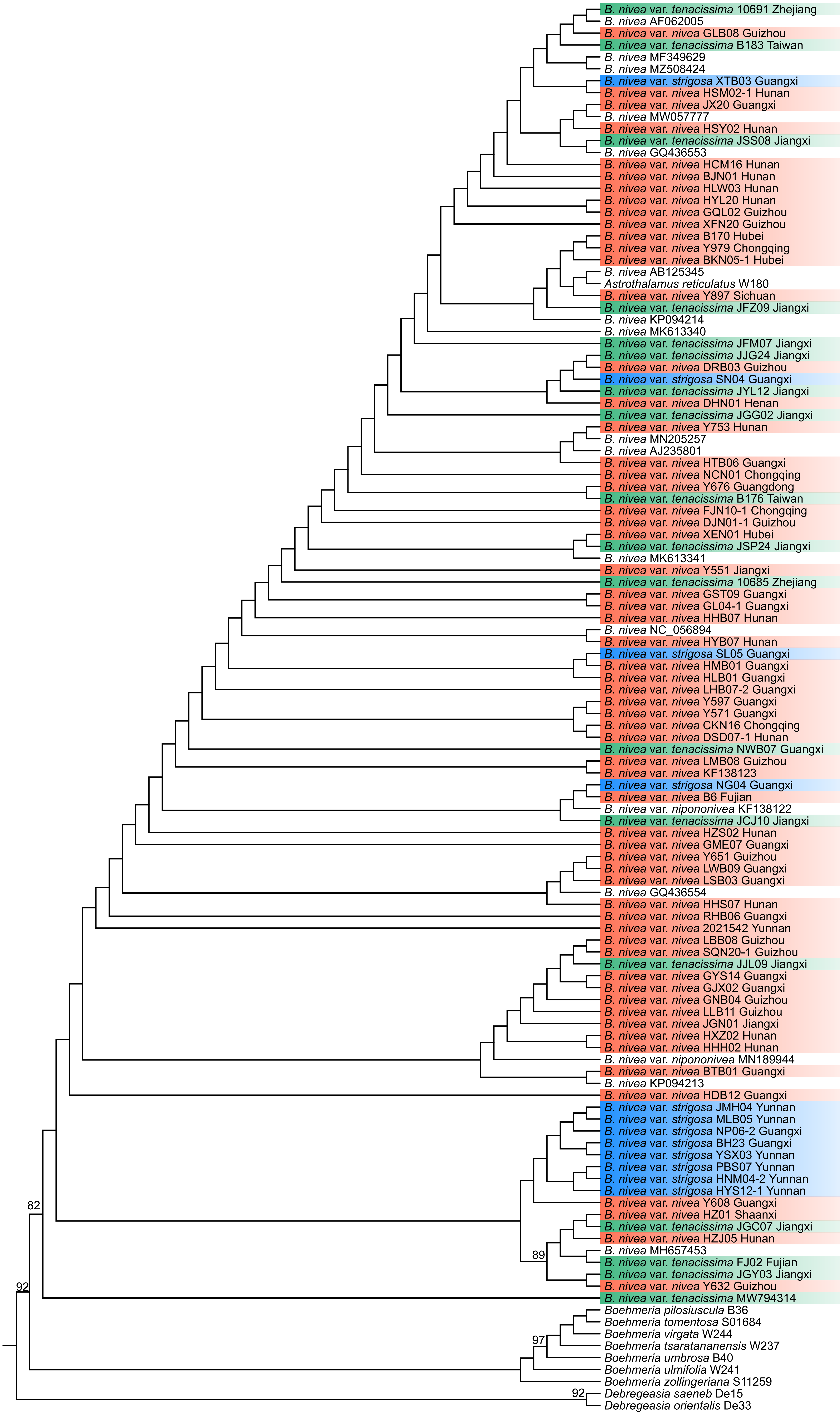

Figure S9. Maximum likelihood phylogenetic tree of *Boehmeria nivea* constructed using *rbc*L dataset (support values only shows ≥ 70%). Each node consists of species name_sample ID_Province except outgroups and downloaded sequences from NCBI.

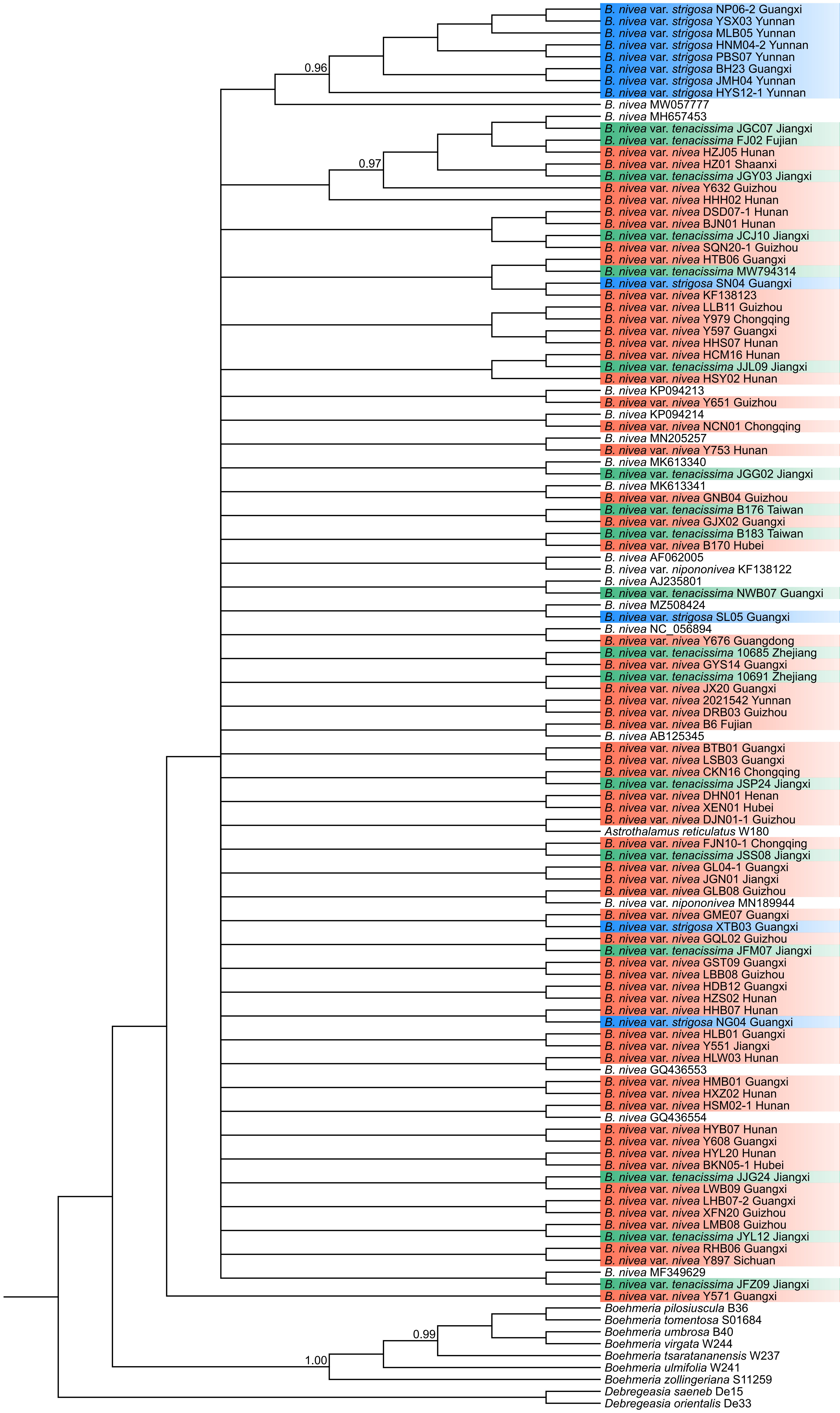

Figure S10. Bayesian phylogenetic tree of *Boehmeria nivea* constructed using *rbc*L dataset (support values only shows ≥ 0.95). Each node consists of species name_sample ID_Province except outgroups and downloaded sequences from NCBI.

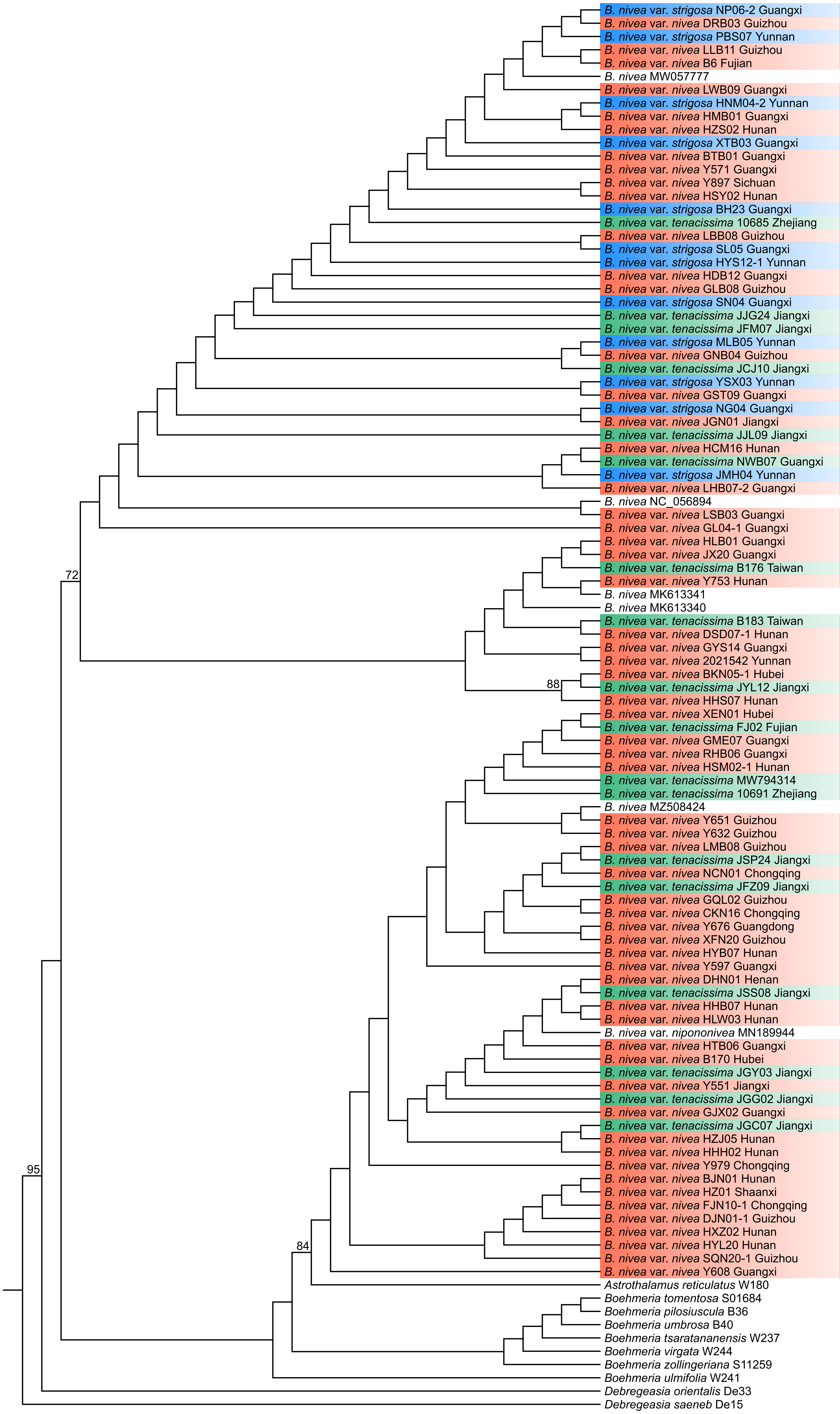

Figure S11. Maximum likelihood phylogenetic tree of *Boehmeria nivea* constructed using *psb*A-*trn*H dataset (support values only shows ≥ 70%). Each node consists of species name_sample ID_Province except outgroups and downloaded sequences from NCBI.

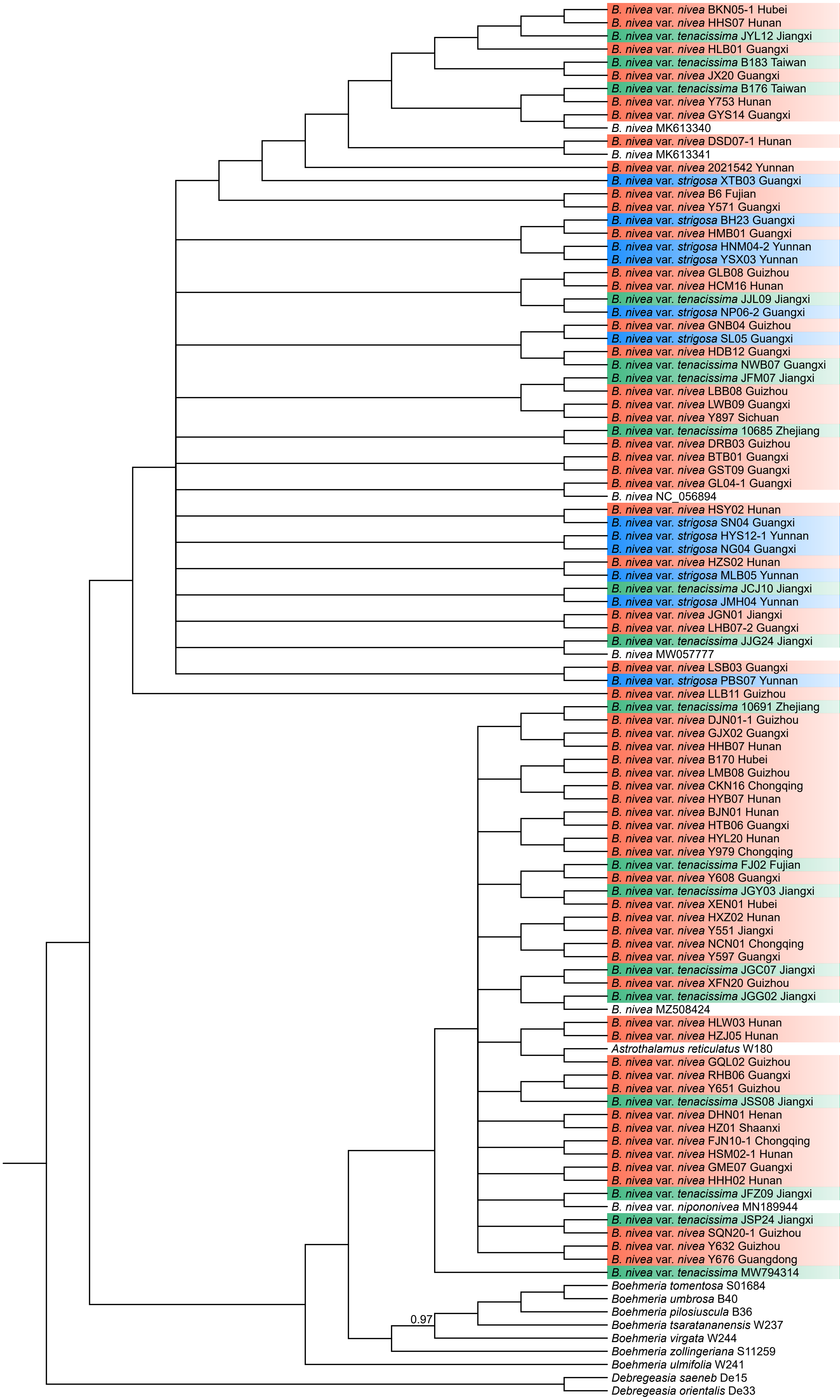

Figure S12. Bayesian phylogenetic tree of *Boehmeria nivea* constructed using *psb*A-*trn*H dataset (support values only shows ≥ 0.95). Each node consists of species name_sample ID_Province except outgroups and downloaded sequences from NCBI.

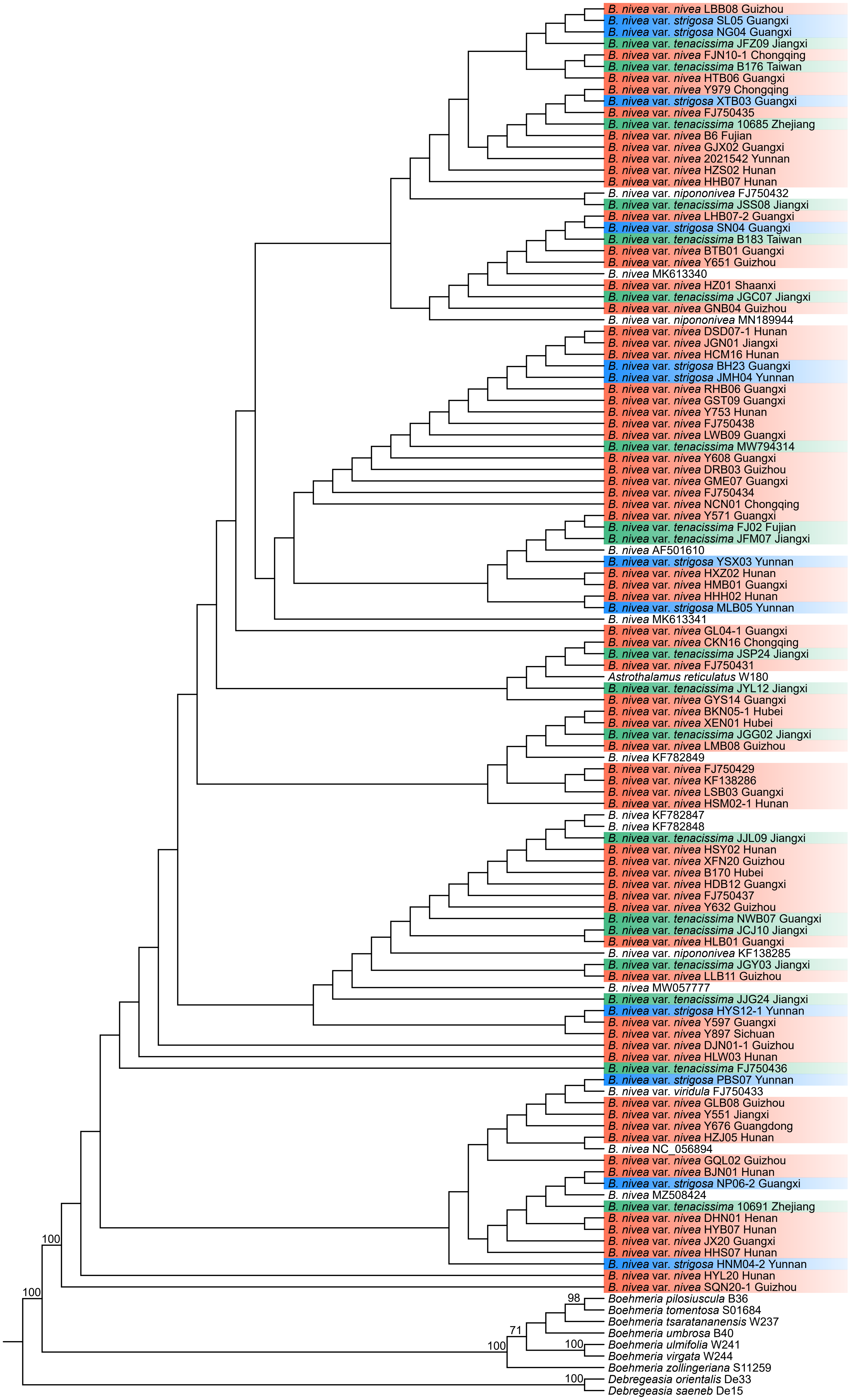

Figure S13. Maximum likelihood phylogenetic tree of *Boehmeria nivea* constructed using *trn*L-*trn*F dataset (support values only shows ≥ 70%). Each node consists of species name_sample ID_Province except outgroups and downloaded sequences from NCBI.

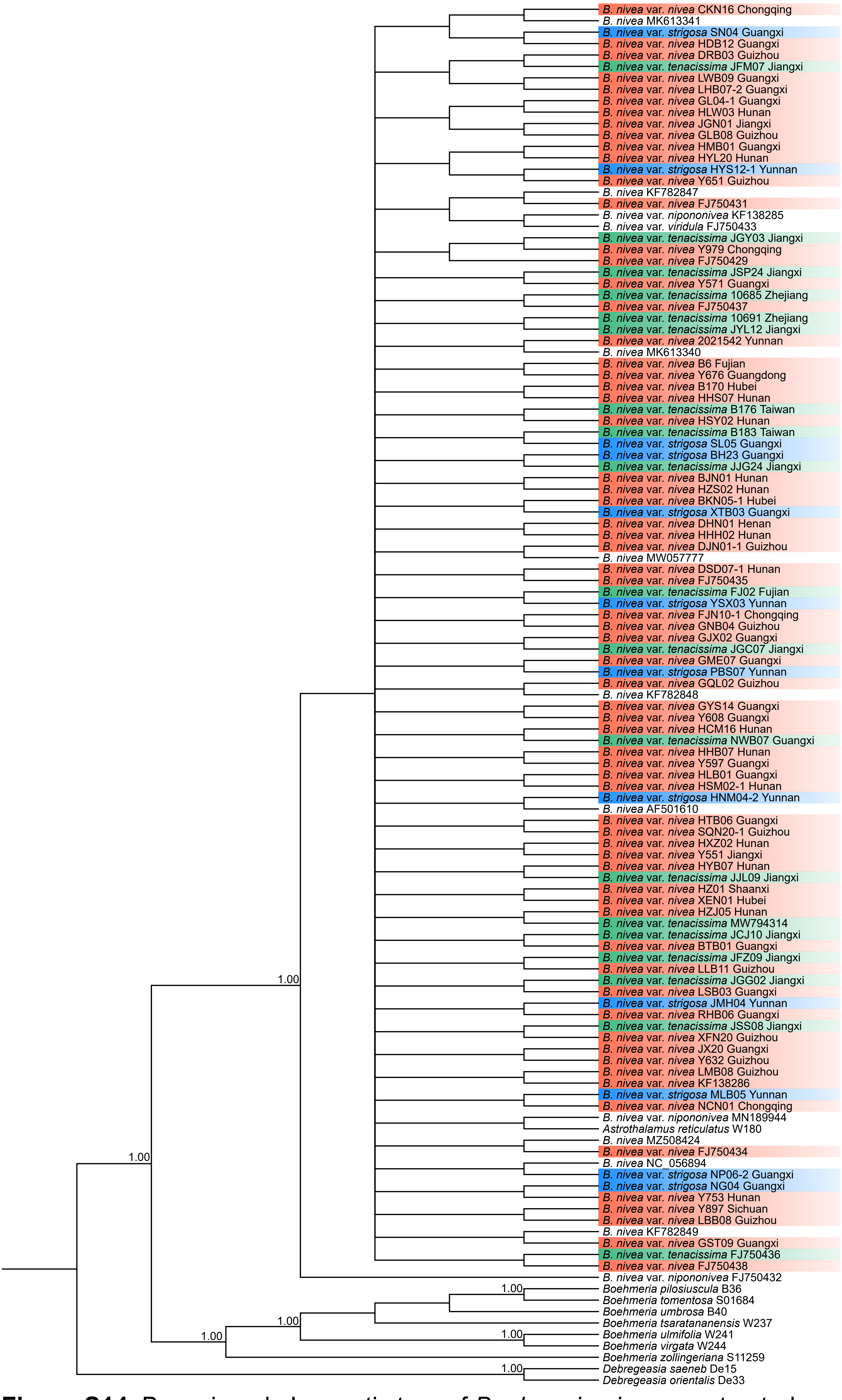

Figure S14. Bayesian phylogenetic tree of *Boehmeria nivea* constructed using *trn*L-*trn*F dataset (support values only shows ≥ 0.95). Each node consists of species name_sample ID_Province except outgroups and downloaded sequences from NCBI.

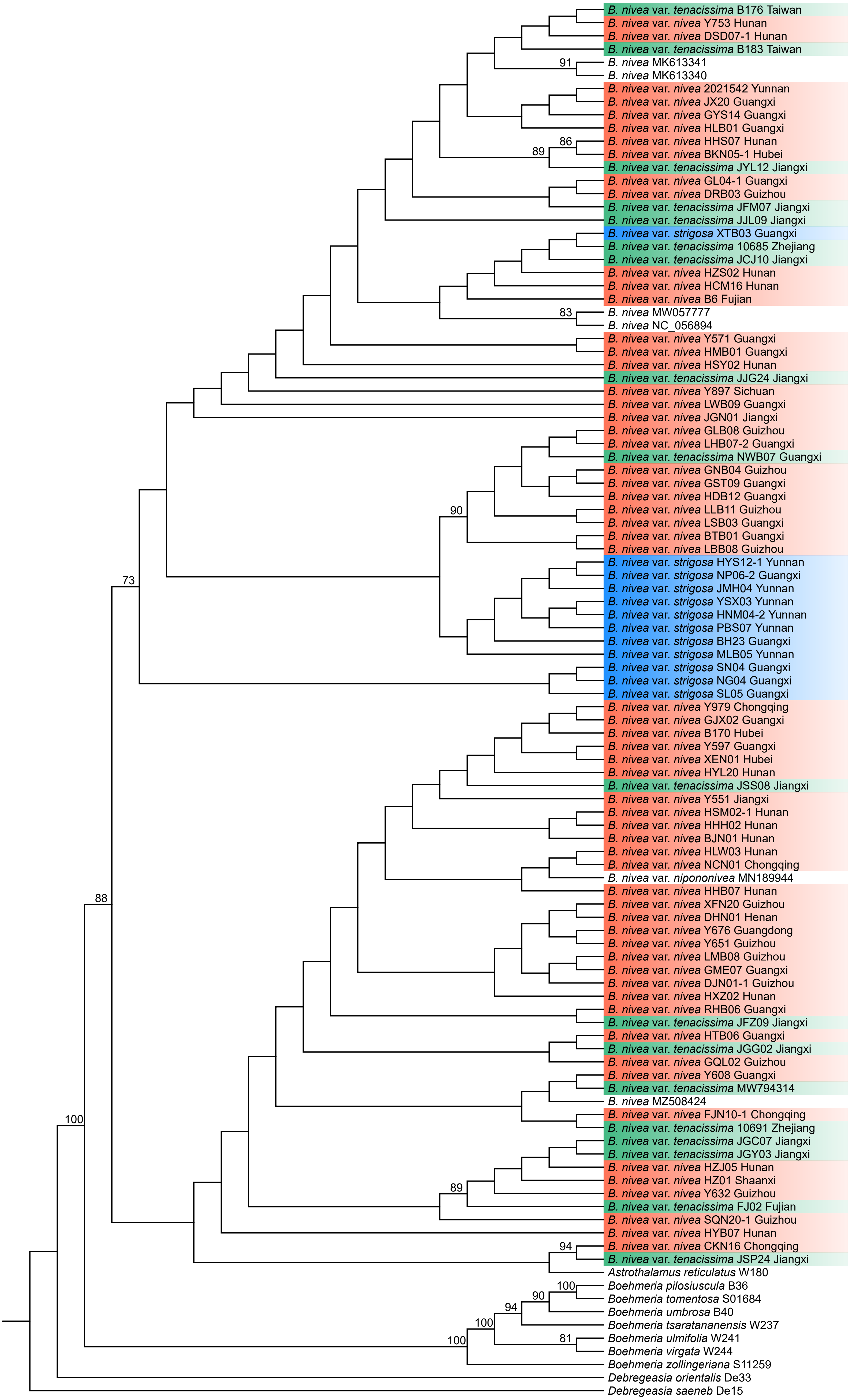

Figure S15. Maximum likelihood phylogenetic tree of *Boehmeria nivea* constructed using *mat*K + *rbc*L + *psb*A-*trn*H + *trn*L-*trn*F dataset (support values only shows ≥ 70%). Each node consists of species name_sample ID_Province except outgroups and downloaded sequences from NCBI.

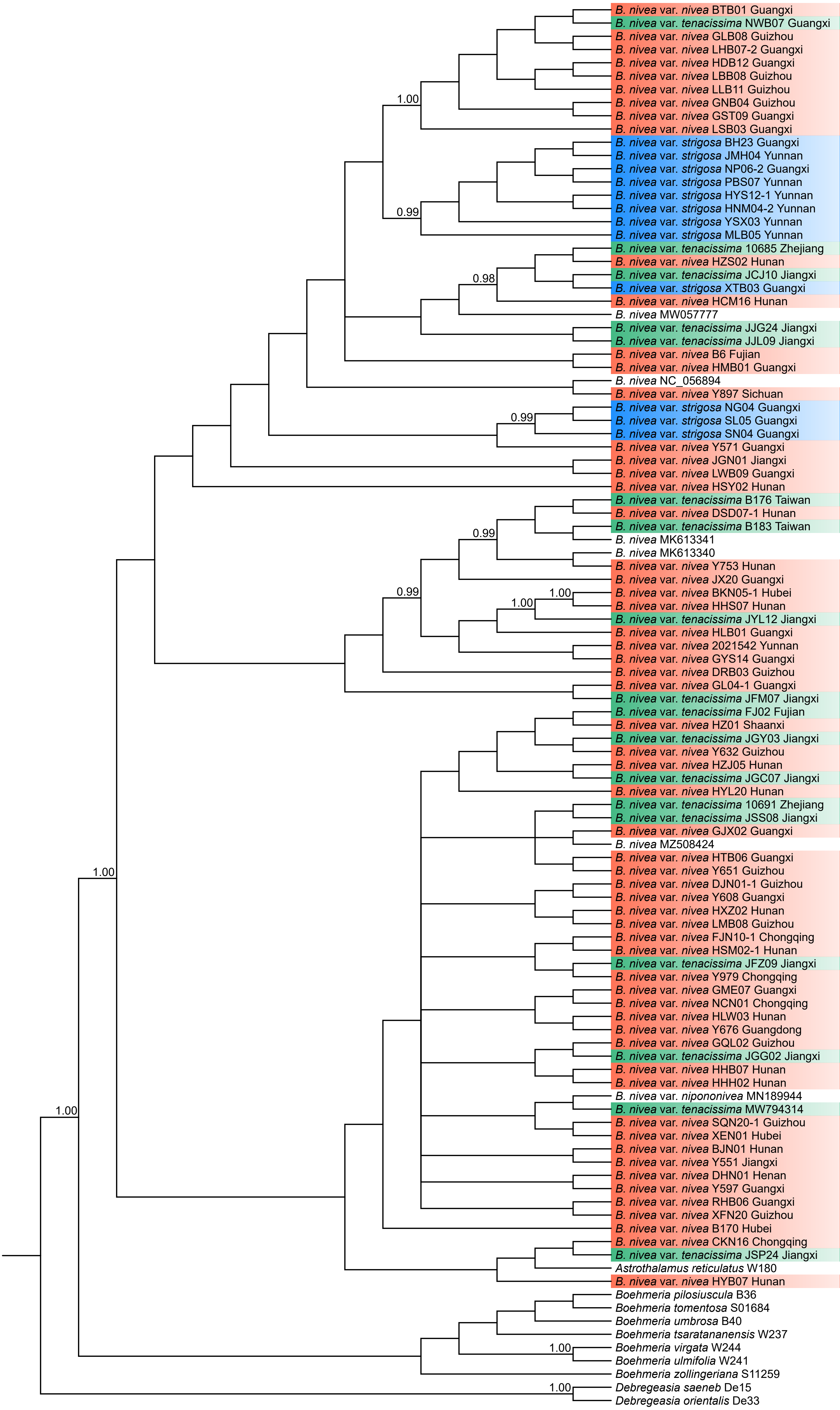

Figure S16. Bayesian phylogenetic tree of *Boehmeria nivea* constructed using *mat*K + *rbc*L + *psb*A-*trn*H + *trn*L-*trn*F dataset (support values only shows ≥ 0.95). Each node consists of species name_sample ID_Province except outgroups and downloaded sequences from NCBI.

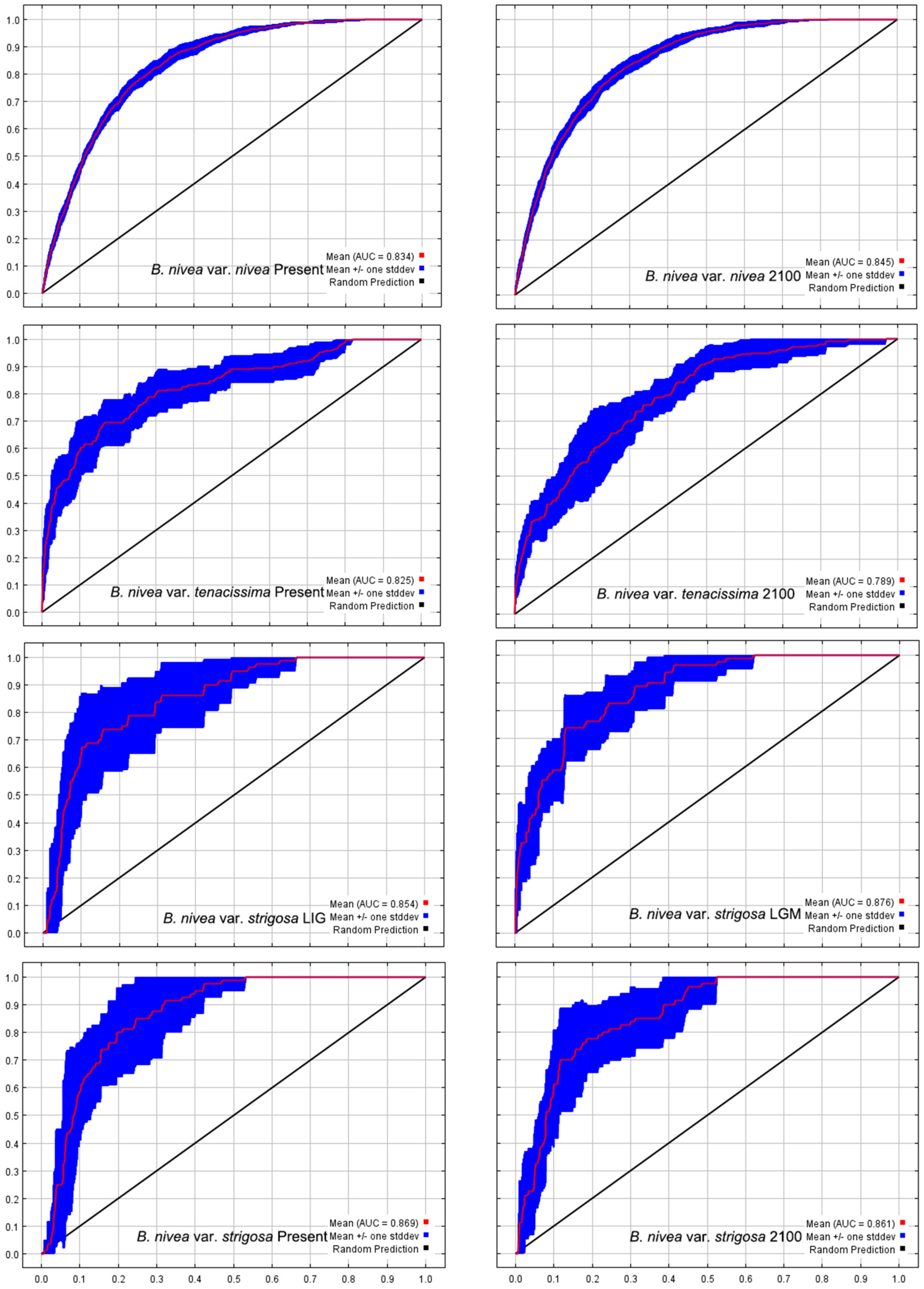

Figure S17. AUC of three variety in *Boehmeria nivea* using ROC methods to test the results of Maxent in different periods.
